# Ouabain-Induced Cell Death and Survival. Role of α1-Na,K-ATPase-Mediated Signaling and [Na^+^]_i_/[K^+^]_i_-Dependent Gene Expression

**DOI:** 10.3389/fphys.2020.01060

**Published:** 2020-09-04

**Authors:** Olga Dmitrievna Lopina, Artem Mikhaylovich Tverskoi, Elizaveta Andreevna Klimanova, Svetlana Vadimovna Sidorenko, Sergei Nikolaevich Orlov

**Affiliations:** ^1^ Department of Biochemistry, Faculty of Biology, Lomonosov Moscow State University, Moscow, Russia; ^2^ Engelhardt Institute of Molecular Biology, Russian Academy of Sciences (RAS), Moscow, Russia; ^3^ Laboratory of Biological Membranes, Faculty of Biology, Lomonosov Moscow State University, Moscow, Russia

**Keywords:** cardiotonic steroids, ouabain, cell death, Na,K-ATPase, intracellular Na^+^ and K^+^, gene expression, intracellular signaling

## Abstract

Ouabain is of cardiotonic steroids (CTS) family that is plant-derived compounds and is known for many years as therapeutic and cytotoxic agents. They are specific inhibitors of Na,K-ATPase, the enzyme, which pumps Na^+^ and K^+^ across plasma membrane of animal cells. Treatment of cells by CTS affects various cellular functions connected with the maintenance of the transmembrane gradient of Na^+^ and K^+^. Numerous studies demonstrated that binding of CTS to Na,K-ATPase not only suppresses its activity but also induces some signal pathways. This review is focused on different mechanisms of two ouabain effects: their ability (1) to protect rodent cells from apoptosis through the expression of [Na^+^]_i_-sensitive genes and (2) to trigger death of non-rodents cells (so-called «oncosis»), possessing combined markers of «classic» necrosis and «classic» apoptosis. Detailed study of oncosis demonstrated that the elevation of the [Na^+^]_i_/[K^+^]_i_ ratio is not a sufficient for its triggering. Non-rodent cell death is determined by the characteristic property of “sensitive” to ouabain α1-subunit of Na,K-ATPase. In this case, ouabain binding leads to enzyme conformational changes triggering the activation of p38 mitogen-activated protein kinases (MAPK) signaling. The survival of rodent cells with ouabain-«resistant» α1-subunit is connected with another conformational transition induced by ouabain binding that results in the activation of ERK 1/2 signaling pathway.

## Na,K-ATP_ase_ as a Target for Cardiotonic Steroids

As back as the 18th century, Withering has published information concerning the therapeutic effect of extracts from the leaves of plants from genus *Digitalis* that were used for the treatment of congestive heart failure by Benedictines (Withering, 1785). Later on this finding led to the isolation of two compounds (digoxin and digitoxin) that were the first found members of plant-derived cardiotonic steroids (CTS) known now as cardenolides (Dmitrieva and Doris, 2002). Besides cardenolides, other members of the CTS family, bufadienolides, have been isolated from amphibians (Krenn and Kopp, 1998). In the end of 20th century, several laboratories demonstrated the presence of compounds identical to cardenolides, namely ouabain (Schneider et al., 1998b; Kawamura et al., 1999), digoxin (Goto et al., 1990), and bufadienolides, such as bufalin (Lichtstein et al., 1993), marinobufagenin (Bagrov and Fedorova, 1998), telocinobufagin (Komiyama et al., 2005), proscillardin A (Schneider et al., 1998a), and 19-norbufalin (Lichtstein et al., 1993), in mammals. Their role in the pathogenesis of hypertension and several other disorders is widely disputed now (Blaustein, 1996; de Wardener, 1996; Lopatin et al., 1999; Dmitrieva and Doris, 2002; Schoner, 2002; Bagrov et al., 2005, 2009; Bagrov and Fedorova, 2005; Khundmiri, 2014; Pavlovic, 2014; Hamlyn and Manunta, 2015; Paczula et al., 2016; Khalaf et al., 2018, 2019; Orlov et al., 2020).

Soon after the discovery of Mg^2+^-dependent (Na^+^,K^+^)-stimulated adenosine triphosphatase (NKA), Skou demonstrated that cardenolide ouabain inhibited the activity of this enzyme (Skou, 1960). Because it was shown earlier (Schatzmann, 1953) that ouabain inhibited active (energy dependent) transport of Na^+^ outside and K^+^ inside the cell, NKA was identified as a system providing for active transport of these cations (Na/K-pump). Now, NKA is considered commonly as the only receptor for CTS, however, discussion concerning the existence of other receptors is continued (Askari, 2019).

NKA is a protein complex of plasma membrane found in almost all animal cells. It consists of ~110 kDa catalytic α-subunit, ~35 kDa β-subunit, and, in most cells studied so far, 8 kDa γ-subunit. It was shown that ATP hydrolysis by NKA is accompanied by the phosphorylation of Asp369 within the active site located on the α-subunit, which provides the E_1_–E_2_ conformational change and electrogenic ion transport (3Na^+^ vs. 2K^+^) with turnover number of 60–80 cycles of phosphorylation-dephosphorylation per second. Besides the ubiquitous α1-isoform, three other α-subunits are expressed in a tissue-dependent manner with high abundance in neuronal tissue (α3 and α2), skeletal muscle, heart (α2), and testis (α4). Four isoforms of β-subunit are highly glycosylated; as a result, their molecular weight is about 55–65 kDa. It was demonstrated that β-subunit participates in the delivery of α-subunit to plasma membrane and affects the affinity of the α-subunit for extracellular potassium (K^+^_o_) and intracellular sodium (Na^+^_i_; Yamaguchi and Tonomura, 1979; Blanco and Mercer, 1998; Geering, 2001, 2008; Rajasekaran et al., 2003). Third NKA subunit that was found in complex with αβ is presented by seven isoforms expressed by tissue-dependent manner. All isoforms sharing a Pro-Phe-X-Tyr-Asp motif (FXYD) and are members of FXYD protein family. This small subunit (7–8 kDa) is a single span membrane protein. It can be bound not only to Na,K-ATPase but also to Na^+^/Ca^+^ exchanger (Cheung et al., 2010). Being bound to NKA, this subunit modulates its function changing the affinity to Na^+^, K^+^, and ATP (Scheiner-Bobis, 2002; Blanco, 2005; Garty and Karlish, 2005; Geering, 2005; Clausen et al., 2017).

The mechanism of NKA inhibition by CTS has been studied mainly with ouabain purified from liana *Strophanthus gratus*. Comparing to other CTS, it has more high solubility in water and is more frequently used in experimental work. Ouabain binds to NKA α-subunit from extracellular space in the deep transmembrane cleft (Ogawa et al., 2009; Shinoda et al., 2009; Yatime et al., 2011; Laursen et al., 2013, 2015). It was shown that transmembrane regions of the α-subunit H1, H5, and H7 and its H1–H2, H5–H6, and H7–H8 extracellular loops affect NKA affinity for ouabain (Lingrel, 2010). It was demonstrated that in rodents, CTS inhibit α1-NKA at concentrations ~10^3^-fold higher than in other mammals. Lingrel and co-workers demonstrated that low affinity of rodent CTS-resistant α1R-NKA to ouabain is caused by the substitution of Gln111 and Asn122 that are in CTS-sensitive α1S-NKA from other mammals, with Arg and Asp, respectively (Lingrel et al., 1997). However, affinity of α2 and α3 isoforms for the CTS in rodents and other mammals is about the same (Lingrel et al., 2007). Taking this into consideration, transgenic mice with α2R‐ and/or α1S-subunits were used to investigate the relative contributions of these isoforms in blood pressure regulation (Dostanic-Larson et al., 2005; Hou et al., 2009), cardiac and skeletal muscle function (Dostanic et al., 2003; Radzyukevich et al., 2009), and renal salt handling (Loreaux et al., 2008).

Treatment cells by CTS affects cellular functions connected with the maintenance of the transmembrane gradient of Na^+^ and K^+^, such as electrical membrane potential (E_m_), cell volume, transepithelial movement of salt and osmotically obliged water, Na^+^(K^+^)/Ca^2+^ and Na^+^/H^+^, symport of Na^+^ with glucose, amino acids, nucleotides, inorganic phosphate, etc. It was shown, during the last two decades, that along with the above-mentioned canonical [Na^+^]_i_, [K^+^]_i_-, E_m_-, and cell volume-mediated cellular responses, CTS can affect gene expression, membrane trafficking, cell adhesion, and proliferation (Xie and Askari, 2002; Aperia, 2007; Liu and Xie, 2010; Riganti et al., 2011; Orlov et al., 2017).

Several groups reported that in contrast to the ubiquitous impact of CTS on Na^+^_i_, K^+^_i_-dependent cell functions, their effects on cell survival are species‐ and tissue-specific. Indeed, 24 h treatment by ouabain induces death of Madin-Darby canine kidney (MDCK) epithelial cells (Ledbetter et al., 1986; Bolívar et al., 1987), endothelial cells from the pig aorta (Orlov et al., 2004b), and different human cells: prostatic smooth muscle cells (Chueh et al., 2001), prostate adenocarcinoma cells (McConkey et al., 2000), monocytes (Kurosawa et al., 2001), erythroleukemia cell line (HEL; Perne et al., 2009), neuroblastoma cell line SH-SY5Y (Kulikov et al., 2007), and neuronal precursor NT2 cells (Hennion et al., 2002; Rosen et al., 2004). In striking contrast, the same treatment has no any impact on the survival of various rat cells: vascular smooth muscle cells (Orlov et al., 1999), endothelial cells, and astrocytes (Akimova et al., 2015), as well as primary cerebellar granule cells (Smolyaninova et al., 2019). We focus our review on mechanisms underlying the distinct impact of CTS on cell survival.

## Ouabain Protects Rodent Cells From Apoptosis *Via* Expression Of [Na^+^]_i_-Sensitive Genes

In all types of cells studied up to date, cell shrinkage is considered as the earliest marker of apoptosis (Bortner and Cidlowski, 1998; Lang and Hoffmann, 2012), particularly in serum-deprived rat vascular smooth muscle cells (RVSMC; Orlov et al., 1996). In addition, similar to most number of studied cells (Matthews and Feldman, 1996; Dmitrieva et al., 2001; Terada et al., 2001; Galvez et al., 2003; Reinehr and Häussinger, 2006; Png et al., 2011; Clouzeau et al., 2012; Burgos et al., 2019), significant shrinkage in hyperosmotic solutions is enough to trigger apoptosis of RVSMC (Orlov et al., 2004a). Because cell volume regulation is mediated by monovalent cations transmembrane gradient (Mongin and Orlov, 2001; Bortner and Cidlowski, 2007; Hoffmann et al., 2009; Pasantes-Morales, 2016; Delpire and Gagnon, 2018), we exposed cells to CTS. Remarkably, we found that practically complete suppression of α1R-NKA activity with 1 mM ouabain prevented RVSMC from apoptosis caused by the removal of growth factor and potentiated by transfection with adenoviral protein E1A (Orlov et al., 1999). To clarify the role of monovalent cations, we expose VSMC to ouabain in high-K^+^, low-Na^+^ medium. We observed that antiapoptotic effect of ouabain entirely abolished by the decrease of the monovalent cations transmembrane gradient (Orlov et al., 1999). These results let us to the conclusion that inhibition of NKA protected RVSMC against apoptosis providing for the increase of the [Na^+^]_i_/[K^+^]_i_ ratio. The antiapoptotic effect of ouabain and K^+^-free medium was found also in a cultured renal proximal tubule cell line (Zhou et al., 2001) and freshly-isolated rat cerebellar granule cells (Isaev et al., 2000). It should be noted that in these investigations, comparatively low ouabain concentrations were used and ouabain action on the activity of NKA and the [Na^+^]_i_/[K^+^]_i_ ratio was not studied.

As will be demonstrated in the next Section, long-term treatment by CTS leads to a decrease of the survival of cultured cells from non-rodent species. Keeping in mind this feature, we inhibited NKA in human umbilical vein endothelial cells (HUVEC) by K^+^-free medium. We detected that Na^+^-containing, K^+^-free medium prevents the development of HUVEC apoptosis due to [^3^H]-decay-induced DNA damage. This protection was not found in low-Na^+^,K^+^-free medium, therefore, we concluded that the antiapoptotic signal is induced by the augmentation of Na^+^_i_ rather than by the attenuation of K^+^_i_ (Orlov et al., 2004b).

**Figure 1:**
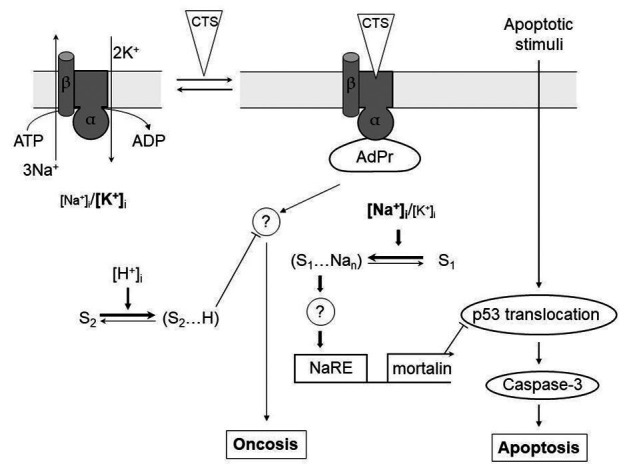
[Na^+^]_i_-mediated and [Na^+^]_i_, [K^+^]_i_-independent signaling pathways involved in the regulation of apoptotic and oncotic modes of cell death by cardiotonic steroids (CTS). α, β – subunits of Na,K-ATPase (different shapes indicate the conformational transition of α-subunit triggered by its interaction with CTS); AdPr – adapter protein interacting with Na,K-ATPase in a CTS-dependent manner; S1 and S2 – hypothetical Na^+^_i_ and H^+^_i_ sensors, respectively; NaRE – Na^+^-response element;? – unknown steps; —–> and —–| – activatory and inhibitory stimuli, respectively. Different letter cases are used to show the different intracellular concentrations of Na^+^ and K^+^. For more details, see the text. Modified from Orlov and Hamet (2006).

To investigate further Na^+^_i_-mediated antiapoptotic pathway, we employed inhibitors of RNA and protein synthesis (actinomycin D and cycloheximide, respectively). Both compounds eliminated protection against apoptosis observed in ouabain-treated RVSMC (Orlov et al., 1999). Taking these data into consideration, later on, we used a proteomics method to recognize Na^+^_i_-sensitive antiapoptotic genes. In RVSMC, ouabain induced expression of 12 soluble proteins. One protein of this set was mortalin, a member of family 70 kDa chaperones, that is involved in regulation of cell division (Taurin et al., 2002b). We determined that RVSMC transfection with mortalin resulted in the inhibition of p53 translocation into the nucleus and similarly to ouabain, retarded the apoptosis development in serum-deprived RVSMC (Taurin et al., 2002b). These results demonstrate that the raise of [Na^+^]_i_ inhibits programmed cell death through increased expression of the mortalin that, in turn, blocks translocation p53 to nucleus caused by the apoptotic stimuli (Orlov and Hamet, 2006). Mechanism of [Na^+^]_i_-mediated pathways leading to the prevention of apoptosis by CTS is presented in Figure 1.

## Search for Na^+^-Sensor(S) Involved in Transcription Of Antiapoptotic Genes

Almost 10-fold elevation of RNA synthesis documented by incorporation of [^3^Н]-uridine in ouabain-treated RVSMC (Orlov et al., 2001) suggests that antiapoptotic effect of NKA inhibition is (at least partially) due to the expression of early response genes (ERGs). Indeed, in RVSMC, we found 10‐ and 4-fold increase of immunoreactive c-Fos and c-Jun after 2‐ and 12-h exposure to ouabain (Taurin et al., 2002a). Later on, employing Affymetrix-based technology, we revealed up to 60-fold changes in the expression of hundreds genes in RVSMC, HUVEC, human adenocarcinoma cell line (HeLa; Koltsova et al., 2012), mouse myoblast C2C12 cell line, rat aorta endothelial cells (RAEC), and primary cultured rat neurones subjected to NKA inhibition by ouabain (Klimanova et al., 2019a). Similar increase of gene expression was observed also in RVSMC, HUVEC, and HeLa in K^+^-free medium that also induced the increase of [Na^+^]_i_/[K^+^]_i_ ratio (Koltsova et al., 2012). Among detected Na^+^_i_/K^+^_i_-sensitive genes that were revealed using two different approaches (ouabain treatment and exposure to K^+^-free medium), 80 transcripts were common (ubiquitous) for all three types of studied cells (Koltsova et al., 2012). Analysis of gene expression induced by ouabain treatment in rodent cells (RVSMC, mouse myoblast C2C12, RAEC, and primary cultured rat neurones) resulted in the finding of 82 ubiquitous Na^+^_i_/K^+^_i_-sensitive genes (Klimanova et al., 2019a).

Approximately 50% of the ubiquitous Na^+^_i_/K^+^_i_-sensitive transcripts were ERGs and other genes involved in the regulation of transcription and/or translation. Thus, we observed more than 3-fold increase of the expression of ubiquitous Na^+^_i_/K^+^_i_-sensitive genes, such as C2H2-type transcriptional regulator of zinc-finger protein *Egr-1*, members of the superfamily of b-Zip transcriptional factors with leucine-zipper motif and basic DNA-binding domain and forming heterodimeric activating protein AP-1 (*Fos*, *FosB*, *Jun*, *JunB*, and *Atf3*), transcription factor of steroid-thyroid hormone-retinoid receptor superfamily *Nr4a2* and basic helix-loop-helix transcription regulator *Hes1* (Taurin et al., 2002a; Haloui et al., 2007; Koltsova et al., 2012; Orlov and Hamet, 2015; Klimanova et al., 2017, 2019a; Orlov et al., 2017). Importantly that 4-fold elevation of *c-Fos* mRNA level was found after 30 min incubation of cells with ouabain. At this time interval, there was ~5-fold [Na^+^]_i_ elevation, whereas [K^+^]_i_ did not changed significantly (Taurin et al., 2002a). These data demonstrate that similar to antiapoptotic effects the EGRs expression is induced by [Na^+^]_i_ raise rather than [K^+^]_i_ loss (Orlov et al., 1999; Taurin et al., 2002a).

In accordance with generally accepted paradigm, gene expression in Na^+^-loaded cells can be affected by changes of [Ca^2+^]_i_ through known signaling pathways. It is assumed that gene expression in this case may be due to an increase in [Ca^2+^]_i_ through the activation of Na^+^/Ca^2+^ exchanger and/or Na^+^-induced depolarization of plasma membrane that results in the opening of voltage-gated Ca^2+^ channels. The rapid elevation of [Ca^2+^]_i_ in the range ~0.1–1 μM results in its binding to calmodulin and other [Ca^2+^]_i_ sensors that affect the genes expression *via* (i) translocation of nuclear factor kappa-light-chain enhancer of activated B cells (NF-κB) from cytosol to the nucleus that is induced by the activation of Ca^2+^/calmodulin-dependent protein kinase (CaMKI, II, or III), (ii) translocation to the nucleus of nuclear factor of activated T-cells (NFAT) triggered by its dephosphorylation by the (Ca^2+^/calmodulin)-dependent phosphatase calcineurin, and (iii) phosphorylation of cAMP response element-binding protein (CREB) by CaMKII and CaMKIV and subsequent its binding to the (Ca^2+^+cAMP)-response element (CRE) sequences of DNA within 5'-untranslated region (5'-UTR; for comprehensive review, see McDonald et al., 1994; Hardingham et al., 1997; Coulon and Blanchard, 2001; Alonso and García-Sancho, 2011; Gundersen, 2011; Ma et al., 2011). Considering that c-Fos promoter includes CRE, its augmented expression in cells treated by ouabain may be mediated by [Ca^2+^]_i_. However, unlike high-K^+^-medium, expression c-Fos in ouabain-exposed cells is not affected by the suppression of L-type Ca^2+^ channels with nicardipine. We also found that augmented c-Fos expression induced by ouabain was preserved in Ca^2+^-free medium and in the presence of extracellular (EGTA) and intracellular (BAPTA) Ca^2+^ chelators (Taurin et al., 2002a; Orlov and Hamet, 2015). Moreover, employing Affymetrix technology it was shown also that Ca^2+^-depletion increased rather than decreased the number of ubiquitous and cell-type specific Na^+^_i_/K^+^_i_-sensitive genes (Koltsova et al., 2012). Among Na^+^_i_/K^+^_i_-sensitive genes whose expression is changed by more than 3-fold independently of the presence of Ca^2+^ chelators, we found *Fos*, *Jun*, *Egr1*, *Atf3*, and several other ERGs. Augmented expression of *Egr1* and *Atf3* in ouabain-treated RVSMC was also preserved in the presence of calmodulin antagonists, as well as inhibitors of CaMK and calcineurin (Koltsova et al., 2012, 2015; Smolyaninova et al., 2017).

All these data together indicate a key role of novel Na^+^_i_-mediated, Ca^2+^_i_-independent mechanisms of excitation-transcription coupling in the antiapoptotic action of CTS documented in rodent cells. Search for Na^+^-sensors among the proteins revealed the presence of Na^+^-selective binding sites in G-protein coupled receptors (GPCR; for recent review, see Klimanova et al., 2019b). This type of G-proteins might be considered as plausible protein Na^+^-sensor.

Examining different regulatory elements of DNA, we turned our attention to so-called G-quadruplexes (GQs), noncanonical secondary structures formed by nucleic acid sequence(s) enriched by guanine bases. GQs are organized from the blocks of the stacking G-quartets each of which consists of four guanine bases assembled in a square planar structure. GQs were found in DNA and diverse types of RNA. In DNA, they are located within various regulatory DNA regions, in particular, in promoters. GQs are present abundantly in genomes of prokaryotes and eukaryotes, suggesting their participation in regulation of gene expression (Ravichandran et al., 2019; Kharel et al., 2020).

The GQs were shown to be stabilized by monovalent cations. They bind to the G-quartets by coordinating to the C6 carbonyl oxygen of the guanines located at or between the plane of the quartets (Balaratnam and Basu, 2015). Cation stabilizing action on GQs depends on their radii and decreases in the following range: K^+^ > Na^+^ and NH_4_^+^ >>> Li^+^ (Włodarczyk et al., 2005). Studies using the HeLa S3 cell line showed that the G4-binding ligand could change the gene expression (Grand et al., 2002). Other authors using the same cell line obtained similar results and found that the promoter regions of differentially expressed genes, including *cMyc*, *cMyb*, and *cFos*, contained G4-forming sequences (Verma et al., 2008).

Considering this information together, we may suggest that GQs (especially GQs located in promoter regions) can affect promoter structure depending on the increase of [Na^+^]_i_/[K^+^]_i_ ratio and, by this way, change the expression of Na^+^_i_/K^+^_i_-sensitive genes. Therefore, GQs might be considered as plausible Na^+^‐ or/and K^+^-sensors within DNA.

However, it should be noted that NKA is considered now as CTS receptor that is working as signal transducer. It was shown that ouabain binding to NKA results in the activation of cytoplasmic tyrosine kinase Src that, in turn, leads to the protein phosphorylation and to the formation of assembles of proteins-partners that are participants of different signaling pathways. This resulted in activation of some genes expression (Xie and Cai, 2003). To discriminate between gene expression that is triggered by the change of [Na^+^]_i_/[K^+^]_i_ ratio or by this type of tissue-specific signaling further experimental studies should be done.

## CTS Trigger Oncosis in Non-Rodent Cells

As noted above, in contrast to RVSMC and other rodent cells, long-term exposure to CTS resulted in the death of cultured cells from human and other non-rodent mammals (McConkey et al., 2000; Chueh et al., 2001; Kurosawa et al., 2001; Hennion et al., 2002; Pchejetski et al., 2003; Rosen et al., 2004; Orlov et al., 2004b; Akimova et al., 2005a, 2015; Kulikov et al., 2007; Pezzani et al., 2014; Meng et al., 2016; Özdemir et al., 2016; Chou et al., 2018). Thus, 24-h exposure to 3 μM ouabain results in the massive death of HUVEC, human aortic smooth muscle cells (HASMC), and human astrocytes, whereas complete inhibition of α1R-NKA by 3 mM ouabain did not affect survival of RVSMC, RAEC, and rat astrocytes. It should be noted that ouabain concentrations used in the study completely inhibited NKA in all types of these cells (Akimova et al., 2015).

Different types of cell death are historically divided into three main types: necrosis, apoptosis, and autophagy. It is worth noting that the morphological characteristics primarily formed the basis of this classification. At the moment, special attention in the description of various types of cell death is given to signal transduction involved in the development of this phenomenon and, therefore, this classification is much more extensive (Galluzzi et al., 2018). It was shown that CTS can induce all three types of death by tissue specific manner (Trenti et al., 2014; Delebinski et al., 2015; Li et al., 2018). In addition, the ability of CTS to induce apopotosis in senescent cells allows to consider them as senolytic compounds. This senolytic activity is mediated by dissipation gradient in Na^+^_i_ and K^+^_i_ as a result of NKA inhibition (Triana-Martínez et al., 2019). Each type of cell death has specific characteristics and specific biochemical markers. For instance, cellular shrinkage is a phenomenon that takes place in apoptosis, cellular swelling is attribute of necrosis, and autophagy is characterized by occurrence of numerous cytoplasmic vesicles. The death of non-rodent cells mentioned above was coupled with cells swelling and their detachment from underlay.

This mode of ouabain-induced cell death terminated by cell swelling and detachment according to morphological properties may be considered as necrosis. But surprisingly, this death was characterized by combined markers of “classic” necrosis (cell swelling, negligible labeling with nucleotides in the presence of terminal transferase, and staining of nuclei with cell-impermeable dyes such as propidium iodide) and “classic” apoptosis (nuclear condensation seen in cells stained with cell-permeable dyes such as Hoechst 33342, chromatin cleavage, and caspase-3 activation; Contreras et al., 1999; Pchejetski et al., 2003; Orlov et al., 2004b). It is interesting to note that there is not only necrosis with marks of apoptosis. It was shown also that apoptosis can occur without cellular shrinkage because shrinkage is mostly due to a decrease in cellular K^+^_i_ content (Yurinskaya et al., 2005). We suggested to use the term “oncosis” (from the Greek word for swelling) just to underline the striking difference in cell volume behavior of CTS-treated non-rodent cells (swelling) vs. shrinkage detected for cells treated with canonical apoptotic stimuli (Majno and Joris, 1995; Van Cruchten and Van den Broeck, 2002; Pchejetski et al., 2003; Orlov and Hamet, 2004; Weerasinghe and Buja, 2012; D’Arcy, 2019). The term “oncosis” was introduced over a century ago by von Recklinghausen and is not used widely today. Oncosis is usually observed in ischemic cells and is characterized by cell swelling, a rapid decrease in the level of intracellular ATP and the inability of cells to maintain an ions gradient, for example, as a result of inhibition of NKA. In addition, this phenomenon occurs faster than apoptosis, and develops within 5 h (Peters et al., 2017).

Several research groups proposed that cell volume increase is sufficient to trigger cell death *via* the plasma membrane rupture (Carini et al., 1995, 1999; Barros et al., 2001; Okada et al., 2001; Piper and Large, 2003). Groulx and co-workers undertook research using dual-image surface reconstruction (DISUR) technique to evaluate the surface area and volume of single substrate-attached cells subjected to severe (6 mOsm) hypotonic stress (Groulx et al., 2006). This study demonstrated that almost all cell types exhibited extremely large membrane reserves and increased their surface area and volume by 4‐ and 10-fold, respectively, mainly through the shape transition drawing extra membrane from pre-existing surfaces and intracellular membrane reserves. We found that 5–10 min before the cell detachment, the volume of ouabain-exposed MDCK cells was increased by ~30–40%. Importantly, we observed also that LDH release began when the volume of hyposmotically-swollen MDCK cells was augmented by ~5-fold. These results show that the rupture of the plasma membrane in ouabain-treated MDCK cells was not induced directly by cell swelling resulted from the NKA inhibition and inversion of the [Na^+^]_i_/[K^+^]_i_ ratio (Platonova et al., 2011).

## CTS-Triggered Oncosis is Mediated by Conformational Transitions of Α1-Subunit

According to the chemo-osmotic model (Carini et al., 1999), cell swelling as a result of NKA inhibition and dissipation of the transmembrane gradients of monovalent cations is sufficient to trigger cell death. Considering this, we examined the role of NKA-mediated ion fluxes in the death of CTS-treated cells by comparing the effect of NKA inhibition by ouabain and K^+^-free medium. Surprisingly, we found that NKA inhibition in K^+^-free medium does not affect the survival of MDCK cells, whereas the addition of ouabain to K^+^-free medium resulted in the same increase of cell detachment and caspase-3 activity as it was detected in control medium (Pchejetski et al., 2003). These data show that dissipation of the transmembrane gradients of monovalent cations and elevation of the [Na^+^]_i_/[K^+^]_i_ ratio is not sufficient for triggering oncosis.

It is known that alongside with “canonical” effects of CTS that are due to the inhibition of NKA and dissipation of Na^+^ and K^+^ gradients, CTS provide for so-called “non-canonical” effects. First of all, we can mention among them regulation by CTS Ca^2+^_i_ concentration through the inhibition of ouabain-sensitive α2-NKA isoform in the heart. Suppression of these NKA isoform increases [Na^+^]_i_ that alters the activity of Na^+^/Ca^2+^ exchanger and, in turn, results in the change in [Ca^2+^]_i_ and force of heart contraction. This action of CTS has been known for many years as the positive inotropic effect (Akera and Brody, 1976). Among “noncanonical” effects, CTS are also ouabain-induced hyperthrophy of heart muscle that is caused by activation of cytoplasmic tyrosine kinase Src and subsequent triggering of signaling resulting in gene expression (Xie and Askari, 2002) and senolytic effects (Triana-Martínez et al., 2019). CTS-induced cell death appears to be in the range of these “non-canonical” effects.

Ward et al. (2002) reported that besides “classic” K^+^_o_-inhibited sites, bovine adrenocortical cells exhibit high-affinity ouabain binding sites in the presence of 20 mM KCl, i.e., under conditions when its binding with NKA is usually negligible. Existence of other receptors for CTS besides NKA once and again is disputed in the literature (for review, see Askari, 2019). However, we did not find high-affinity ouabain-binding sites in C7-MDCK exposed to high-K^+^ medium (Akimova et al., 2005a). Moreover, the same left-hand shift was noted in comparison to the dose-dependent action of ouabain on Na^+^/K^+^-pump activity and death of C7-MDCK and PAEC (Pchejetski et al., 2003; Orlov et al., 2004b). The data strongly suggest that in both cell types, CTS trigger Na^+^_i_, K^+^_i_-independent oncosis through the binding to the NKA α-subunit rather than through another potential K^+^_o_-insensitive receptor. In accordance with this, we suggest that CTS-induced conformational transition of the NKA α-subunit, which was first described by Jorgensen (Hegyvary and Jorgensen, 1981) is sufficient to induce interaction of α-subunit with unidentified adapter protein (AdPr), which, in turn, triggers signaling pathway resulting in Na^+^_i_, K^+^_i_-independent oncosis of renal epithelial and vascular endothelial cells (Figure 1).

Importantly, various CTS differentially affect Na^+^_i_, K^+^_i_-mediated and independent signaling. Thus, marinobufagenin and marinobufatoxin raised [Na^+^]_i_ in MDCK cells at the same concentration range as ouabain but these bufadienolids were much less potent in triggering Na^+^_i_, K^+^_i_-independent oncosis (Akimova et al., 2005a). It was shown also that infusion of marinobufagenin increased cardiomyocyte apoptosis through activation of the Src/Akt/mTOR signaling pathway (Liu et al., 2012).

**Figure 2:**
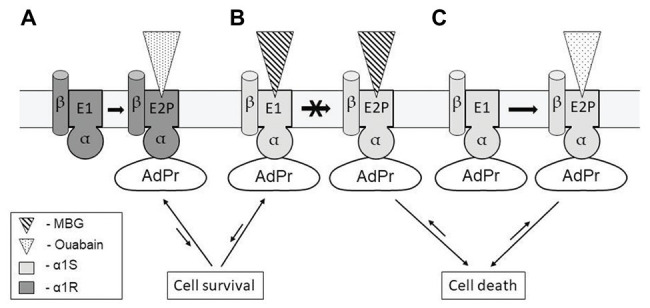
Possible mechanisms representing the absence of cell death signaling due to ouabain binding to α1R-NKA **(A)**; and the appearance of this signaling with distinct potency as result of marinobufagenin **(B)** and ouabain binding to α1S-NKA **(C)**. Conformational transition induced by ouabain binding to α1R-NKA does not affect the binding of unidentified adaptor protein (AdPr) to NKA **(A)**. Marinobufagenin (MBG) with similar affinity freezes α1S-NKA in conformations E1 and E2P (transition between them is prohibited), AdP is released from complex with E1-conformation providing cell survival and does not release from complex with E2P providing cell death **(B,C)**. For more details, see the text.

It is known that binding of ouabain and marinobufagenin to NKA α-subunit freezes the enzyme in two distinct conformations. We have shown earlier that in contrast to ouabain that binds to NKA only in E2P conformation (*K*_d_ value 0.1 μM) marinobufagenin binds to both E1 and E2P conformations of the enzyme with similar affinity (*K*_d_ values equal to 3.6 and 1.7 μM, respectively; Klimanova et al., 2015). To explain various effects of ouabain and marinobufagenin on cell death, we propose that NKA α-subunit tightly binds the unidentified AdPr to E1, thus providing cell survival and unable to generate any additional Na^+^_i_, K^+^_i_-independent signals (Figure 2A). In E2P-conformation with bound CTS (ouabain or marinobufagenin), AdPr dissociates from NKA and triggers Na^+^_i_, K^+^_i_-independent signaling resulting in cell death (Figure 2B,C). Because marinobufagenin freezes NKA in both conformations (E1 and E2P) with similar affinity, the amount of complex E2P-MBG and concentration of cytoplasmic AdPr (that triggers cell death signaling) is less and insufficient to provide signaling at concentrations comparable with concentrations of ouabain. To prove this model, further studies should be performed.

## Rodent Α1R-NKA and Cytosol Acidification Rescue Cells From Cytotoxic Action of Ouabain

Mechanisms underlying the species‐ and tissue-specific effects of CTS on cell death and survival remain poorly understood. As we mentioned above, in rodent cells, ouabain inhibits ubiquitous α1-NKA at concentrations up to three orders of magnitude higher than in other mammals. This phenomenon is due to mainly the substitution of two amino acids in the polypeptide chain of NKA α1-subunit in ouabain binding site (Gln111 and Asn122 detected in CTS-sensitive mammalian α1S-NKA are changed by Arg and Asp in CTS-resistant rodent α1R-NKA, respectively; Lingrel et al., 1997). However, despite the exposure of rodent cells to high ouabain concentrations that was sufficient to inhibit NKA completely and to change the [Na^+^]_i_/[K^+^]_i_ ratio in rodent cells to the same extent as in other mammalian cells, rodent cells survive.

To examine specific role of NKA α1-subunits, MDCK cells that express α1S were stably transfected with α1R-NKA (Akimova et al., 2010b). Treatment of α1R-cells with 1 mM ouabain for 6 h led to similar increment of the [Na^+^]_i_/[K^+^]_i_ ratio that was found in mock-transfected cells treated with 3 μM ouabain. However, in contrast to the massive death of mock-transfected cells exposed to 3 μM ouabain, α1R-cells survived after 24-h incubation with 1 mM ouabain (Akimova et al., 2010b). Based on this finding, we proposed that the α1R-subunit rescues cells from the cytotoxic action of CTS independently of the suppression of Na^+^ and K^+^ fluxes mediated by NKA. We postulated that the cytotoxic effect of α1S on rodent cells is caused by its specific conformational changes induced by CTS binding. According to this model (Figure 2B), CTS (like ouabain) induce dissociation (or association) of α1S and unidentified AdPr, triggering the cell death signal, whereas its interaction with α1R is preserved in the presence of CTS (Akimova et al., 2009). Our data obtained in the experiments with restricted trypsinolysis of purified NKA from pig (α1S-NaKA) and rat (α1R-NKA) kidney treated by ouabain demonstrated that the sets of α-subunit proteolytic fragments of these two enzymes were different (Tverskoi et al., 2020). This finding supports the idea that ouabain binding to α1R‐ and α1S-NKA induces distinct enzyme conformations. All data together indicate that the death of non-rodent cells exposed to ouabain is provided for a signal that is triggered as result of CTS binding to α1S‐ but not to α1R-NKA. Namely this specific transition of α1S-NKA conformation appears to evoke the interaction of enzyme with unknown AdPr, inducing signaling pathway leading to cell death. These data are in agreement with Blaustein and Hamlyn conclusion (Blaustein and Hamlyn, 2020). Considering the functions of NKA isoforms in rodent, they decided that ubiquitous α1-NKA is mainly responsible for the maintaining the Na^+^_i_ and K^+^_i_ gradients, while the signaling functions of ouabain in rodent cells are realized through their interaction with α2‐ and α3-NKA.

Another way for preventing ouabain-induced death in non-rodent mammalian cells has also been disclosed. Analyzing the role of extracellular ions in CTS-induced oncosis, we found that a decrease of NaHCO_3_ concentration from 44 to 11 mM strongly reduced the death of C7-MDCK cells induced by ouabain. Further experiments demonstrated that medium acidification that was provided for a decreased HCO_3_^−^/CO_2_ ratio was sufficient to rescue the cells. Inhibition of cell death was found in HEPES acidified medium with high concentration of NaHCO_3_. Whereas, the increase in pH caused by the addition of Tris eliminated the protective effect of depletion of NaHCO_3_ (Akimova et al., 2006). Using NaHCO_3_-free, HEPES-tris-buffered medium, we revealed that the death of ouabain-treated PAEC and C7-MDCK cells is inhibited by the acidification of the medium from pH 7.4 to 7.0 (pH inside the cell was decreased from ~7.2 to 6.9). The survival of ouabain-treated C7-MDCK cells was also observed as result of selective intracellular acidification induced by the suppression of Na/H exchanger with ethylisopropyl amiloride (Akimova et al., 2006). Influence of acidification on cell death induced by CTS may be due to the protonation of amino acid residues of some unknown protein(s) affecting the cell death signaling pathway (it is schematically presented in Figure 1). It also cannot be excluded that these unknown protein(s) can affect the concentration of some cations in cytosol that, in turn, disrupts the signal transduction resulting in cell death. It is known, for example, that acidification inhibits TASK-3 K^+^ channels (Rajan et al., 2000) but activates TREK-1 K^+^ channels (Maingret et al., 1999) and Ca^2+^-permeable acid-sensitive ion channels (Xiong et al., 2004). However, the change of these transporters activity was observed in pH range 7.4–5.0 that corresponds to ~200-fold elevation of [H^+^]_i_ concentration and is in contrast with the ~3-fold [H^+^]_i_ increase that is sufficient for complete inhibition of cell death signaling (Akimova et al., 2006).

Modest acidification did not change [^3^H]-ouabain binding and ouabain-sensitive ^86^Rb inflow (Akimova et al., 2006), showing that the pH-sensitive component of the cell death mechanism is located downstream of NKA. It should be noted also that a pH decrease from 7.2 to 5.0 activates caspases (Matsuyama and Reed, 2000) and nucleases (Pérez-Sala et al., 1995). Thus, such enzymes may be excluded as a possible pH-sensor involved in the inhibition of death signaling pathway triggered by CTS.

Elongation factor-2 (eEF-2) is the conspicuously phosphorylated protein that was found in the extracts of mammalian tissue. It was shown that the phosphorylation of this protein by the eEF-2 kinase results in the protein synthesis inhibition (Ryazanov et al., 1988). Comparison of liver homogenate from wild-type and eEF-2 kinase knockout mice demonstrated that the elevation of pH from 6.6 to 7.4 (i.e., in the range of pH where regulation of the CTS-induced cell death mechanism was detected) fully inhibits eEF-2 phosphorylation [Dorovkov et al. (2002); Ryazanov (2002)]. Taking this into consideration, it may be suggested that acidosis inhibits the signal of cell death through the inhibition of protein synthesis by eEF-2 phosphorylation. Nevertheless, well-known inhibitors of RNA and protein synthesis did not inhibit the death of ouabain-exposed MDCK, whereas the protective action of acidification was strongly suppressed by these drugs at low non-toxic doses (Akimova et al., 2006). These data assume that the protection from Na^+^_i_, K^+^_i_-independent oncosis induced by CTS in renal epithelial and vascular endothelial cells is mediated by the *de novo* expression of gene(s) involved in the inhibition of the cell death machinery.

Considering physiological importance of these phenomena, we should noted that acidification of cytosol pH lower 6.5 is a marker of hypoxia and ischemia (Siesjö et al., 1996). In some tissues, like heart (Williams and Benjamin, 2000), brain (Barone et al., 1998), and kidney (Islam et al., 1997), brief ischemic preconditioning prevents cells from death that is induced by acute ischemia (Lee et al., 2000). It is interesting, that like ouabain-treated cells, the protective effect of ischemic preconditioning on tissue injury induced by acute ischemia is transient (Li et al., 1992). This effect is also significantly decreased by cycloheximide (Barone et al., 1998). Moreover, the protective effect of acidification is not restricted by CTS-treated cells but also was detected in serum-deprived bovine and HUVECs (D’Arcangelo et al., 2000, 2002) and endothelial cells from human pulmonary arteries after its exposure to staurosporine (Cutaia et al., 2000).

Unfortunately, we do not understand now precise mechanisms underlying cytotoxic effect of CTS resulting in oncosis of non-rodent mammalian cells and its prevention by modest acidosis. We can only note the following specific features of this fenomena: (1) oncosis is due to the conformational transition of NKA that is induced by CTS binding to α1S‐ but not to α1R-NKA, (2) an intermediate participating in the prevention of CTS induced cell death through the modest cytosol acidification is located downstream the NKA and is a result of *de novo* expression of some gene(s), and (3) this effect is not connected with the change of the activity of some known pH-dependent transporters of K^+^ and Ca^2+^ and some pH-dependent enzymes like caspases or nucleases.

## Search for Ouabain-Specific Downstream Signaling Pathways

Thus, at this time, the precise mechanism, by which ouabain triggers signaling pathway of cell death, is not clear. However, numerous findings suggest NKA as an important signal transducer. Direct protein interactions between the NKA and its protein partners should be responsible for this newly signaling mechanism.

To study further the downstream signaling pathway, it is necessary to identify AdPr, which interacts with α1S-NKA or dissociates from it as result of enzyme conformational transition induced by ouabain. The NKA α-subunit is known to interact with various cellular proteins, including proteins of the cytoskeleton network (Morrow et al., 1989; Koob et al., 1990; Kraemer et al., 1990, 2003; Lee et al., 2001), Src kinase (Haas et al., 2002), clathrin (Liu et al., 2004), adaptor protein 2 (AP-2; Liu et al., 2004), caveolin-1 (Wang et al., 2004), inositol 1,4,5-triphosphate (InsP_3_) receptor type 3 (Miyakawa-Naito et al., 2003), and the regulatory subunit (p85α) of class I_A_ phosphoinositide-3 kinase (PI3K; Yudowski et al., 2000). Futhermore, it was shown that in LLC-PK1 and COS-7 cells treated by ouabain, the interaction of NKA with Src kinase (Haas et al., 2002), caveolin-1 (Wang et al., 2004), clathrin (Liu et al., 2004), AP-2 (Liu et al., 2004), and InsP_3_ receptor is increased (Miyakawa-Naito et al., 2003).

We may ask: are these proteins upstream adaptors in CTS-exposed cells that activate signaling pathway resulting in oncosis? We did not observe any impact of the wortmannin (PI3K inhibitor) on the death of ouabain-treated C7-MDCK cells. Negative results were also found with employment of Ca^2+^ chelators (both extra‐ and intracellular) and the compounds increasing Ca^2+^_i_ concentration (Akimova et al., 2005b). It was shown also (using method of fluorescent resonance energy transfer) that interactions between NKA and InsP_3_ receptor are terminated after disruption of actin microfilaments after treatment with cytochalasin D. Cytochalasin and vinblastin (an irreversible inhibitor of microtubule polymerization) also did not abolish the death of C7-MDCK cells exposed to ouabain (Akimova et al., 2005b). These data do not confirm ubiquitous role of clathrin, InsP_3_ receptor, the PI3K regulatory subunit, and AP-2 as upstream AdPr participating in the triggering of oncosis in ouabain-treated cells.

To find unknown AdPr using proteomic approach, Caco-2 human colorectal adenocarcinoma cells were exposed to ouabain (3 μM) for 3 h (21 h before the death) and proteins interacting in complex with the NKA were coimmunoprecipitated from cell lysate using antibodies against α1-subunit. These proteins were separated by 2D electrophoresis and analyzed by mass spectroscopy. We revealed more than 20 proteins with molecular masses in the range 33–71 kDa whose interaction with NKA α-subunit was activated by ouabain. Besides some mentioned above proteins, we identified also seven additional proteins. Some of these proteins may be considered as participants of signal pathways. This set of proteins consisted of α-isoform of protein phosphatase 2C, Rac-GTPase activating protein 1, Src-kinase associated phosphoprotein 1, serum/glucocortocoid regulated kinase 2, glucocorticoid receptors, and casein kinase 1 σ (Akimova et al., 2016). The role of these proteins in CTS-induced Na^+^_i_, K^+^_i_-independent death signaling should be examined further.

It is well known that various mitogen-activated protein kinases (MAPK) has different effects on cell survival and death (Boutros et al., 2008; Thornton and Rincon, 2009; Kim and Choi, 2010). Phosphorylation of extracellular signal-regulated kinase (ERK) plays an important role in the regulation of cell proliferation and differentiation. In contrast, the phosphorylation of c-Jun N-terminal kinase (JNK) and p38 MAPK is often associated with stress, inflammation, and activation of these kinases is connected with cell death (Munshi and Ramesh, 2013; Li et al., 2014). To detect what type of MAPK is associated with death and survival of cells exposed to ouabain, we studied their activation after the addition of ouabain to cells. It was found that phosphorylation of p38 MAPK precedes oncosis of the ouabain-treated MDCK cells (Akimova et al., 2009, 2010a). Comparison of ouabain effect on the phosphorylation of MAPKs in HUVEC and RAEC showed that 6-h incubation with ouabain resulted in ~2‐ and 5-fold elevation of ERK1 and ERK2 phosphorylation in RAEC but with very modest changes in their phosphorylation in HUVEC. In this study, we also detected ~2-fold ouabain-induced increase in p38 phosphorylation in HUVEC without any changes in RAEC. We did not find any significant change of the phosphorylation of JNK1/2 MAPKs in both types of cell (Akimova et al., 2015).

Overall, our results demonstrated that in rodent cells, ouabain is not toxic, possibly because the signaling cascade triggered by its interaction with α1R-NKA results in protective activation of ERK 1/2. In contrast, in HUVEC and MDCK cells expressing α1S-NKA, it activates p38 MAPK.

## Conclusion and Unresolved Issues

Finally, we may conclude that CTS can induce both non-rodent cell death and rodent cell survival. Study of these two impacts of CTS reveals the following:

1. Ouabain is capable to protect RVSMCs from apoptosis that is triggered by growth factor withdrawal, this effect is due to the increase of [Na^+^]_i_/[K^+^]_i_ ratio that, in turn, results in the inducing of [Na^+^]_i_-sensitive genes expression. Among proteins that are encoded by [Na^+^]_i_-sensitive genes is mortalin, a member of family 70 kDa chaperones, which inhibits p53 translocation into the nucleus and prevents the development of apoptosis. Key role in prevention of apoptosis of rodent cells plays novel Na^+^_i_-mediated, Ca^2+^_i_-independent mechanisms of excitation-transcription coupling. Nature of Na^+^-sensor(s) is unknown now. However, noncanonical secondary structures formed by nucleic acid sequence(s) enriched by guanine bases (so called G-quadruplexes) may be considered as a candidate for role of Na^+^-sensor(s) within DNA.
2. CTS are capable induce cell death of different mammalian cells but not rodent cells. This death has combined hallmarks of apoptosis and necrosis and was called oncosis. The increase of the [Na^+^]_i_/[K^+^]_i_ ratio is not sufficient for triggering oncosis, it is produced by CTS binding to α1S‐ but not to α1R-subunit of NKA. This is due to the characteristic property of α1S-NKA and its ability to specific ouabain-induced conformational change that was not found for α1R-NKA. Another way to prevent ouabain-induced death in non-rodent mammalian cells is acidification of medium from pH 7.4 to 7.0. Downstream intermediates of signaling pathway from α1S-NKA that trigger death machinery are unknown now. However, this pathway results in the activation of p38, in contrast to rodent cells where the binding of ouabain to α1R-NKA activates ERK 1/2 that results in cell survival.

Several key questions should be answered to understand the mechanisms of the tissue-specific effect of CTS and NKA on cell survival and death. What is [Na^+^]_i_-sensor(s) and Na^+^-response element involved in the expression of genes providing for antiapoptotic effect? Why different CTS induce different conformation changes of αS-NKA? Which AdPrs interact(s) with α-NKA in a CTS-dependent manner, providing for triggering of oncotic signal and what are intermediates of this cascade? Which elements of this signaling cascade are missing in rodent cells surviving in the presence of CTS? What is pH_i_ sensor affecting the [Na^+^]_i_/[K^+^]_i_-independent oncosis? Does this sensor participate in the phenomenon of ischemic preconditioning? We will address these questions in forthcoming studies.

## Author Contributions

All authors listed have made a substantial, direct, and intellectual contribution to the work, and approved it for publication.

### Conflict of Interest

The authors declare that the research was conducted in the absence of any commercial or financial relationships that could be construed as a potential conflict of interest.

## References

[ref1] Akera T. Brody T. M. (1976). Inotropic action of digitalis and ion transport. Life Sci. 18, 135–141. 10.1016/0024-3205(76)90017-5, PMID: 130524

[ref2] Akimova O. A. Bagrov A. Y. Lopina O. D. Kamernitsky A. V. Tremblay J. Hamet P.. (2005a). Cardiotonic steroids differentially affect intracellular Na^+^ and [Na^+^]_i_/[K^+^]_i_-independent signaling in C7-MDCK cells. J. Biol. Chem. 280, 832–839. 10.1074/jbc.M411011200, PMID: 15494417

[ref3] Akimova O. A. Kapilevich L. V. Orlov S. N. Lopina O. D. (2016). Identification of proteins whose interaction with Na^+^,K^+^-ATPase is triggered by ouabain. Biochem. Mosc. 81, 1013–1022. 10.1134/S0006297916090108, PMID: 27682173

[ref4] Akimova O. A. Lopina O. D. Hamet P. Orlov S. N. (2005b). Search for intermediates of Na^+^,K^+^-ATPase-mediated [Na^+^]_i_/[K^+^]_i_-independent death signaling triggered by cardiotonic steroids. Pathophysiology 12, 125–135. 10.1016/j.pathophys.2005.03.003, PMID: 16023561

[ref5] Akimova O. A. Lopina O. D. Rubtsov A. M. Gekle M. Tremblay J. Hamet P.. (2009). Death of ouabain-treated renal epithelial cells: evidence for p38 MAPK-mediated Na_i_^+^/K_i_^+^-independent signaling. Apoptosis 14, 1266–1273. 10.1007/s10495-009-0404-0, PMID: 19784777

[ref6] Akimova O. A. Lopina O. D. Rubtsov A. M. Hamet P. Orlov S. N. (2010a). Investigation of mechanism of p38 MAPK activation in renal epithelial cell from distal tubules triggered by cardiotonic steroids. Biochem. Mosc. 75, 971–978. 10.1134/s0006297910080043, PMID: 21073417

[ref7] Akimova O. A. Pchejetski D. Hamet P. Orlov S. N. (2006). Modest intracellular acidification suppresses death signaling in ouabain-treated cells. Pflugers Arch. 451, 569–578. 10.1007/s00424-005-1493-4, PMID: 16052351

[ref8] Akimova O. A. Tremblay J. Van Huysse J. W. Hamet P. Orlov S. N. (2010b). Cardiotonic steroid-resistant alpha1-Na^+^,K^+^-ATPase rescues renal epithelial cells from the cytotoxic action of ouabain: evidence for a Na_i_^+^,K_i_^+^-independent mechanism. Apoptosis 15, 55–62. 10.1007/s10495-009-0429-4, PMID: 19949978

[ref9] Akimova O. A. Tverskoi A. M. Smolyaninova L. V. Mongin A. A. Lopina O. D. La J.. (2015). Critical role of the α1-Na^+^,K^+^-ATPase subunit in insensitivity of rodent cells to cytotoxic action of ouabain. Apoptosis 20, 1200–1210. 10.1007/s10495-015-1144-y, PMID: 26067145 PMC4677477

[ref10] Alonso M. T. García-Sancho J. (2011). Nuclear Ca(2+) signalling. Cell Calcium 49, 280–289. 10.1016/j.ceca.2010.11.004, PMID: 21146212

[ref11] Aperia A. (2007). New roles for an old enzyme: Na,K-ATPase emerges as an interesting drug target. J. Intern. Med. 261, 44–52. 10.1111/j.1365-2796.2006.01745.x, PMID: 17222167

[ref12] Askari A. (2019). The sodium pump and digitalis drugs: dogmas and fallacies. Pharmacol. Res. Perspect. 7:e00505. 10.1002/prp2.505, PMID: 31360524 PMC6639696

[ref13] Bagrov A. Y. Fedorova O. V. (1998). Effects of two putative endogenous digitalis-like factors, marinobufagenin and ouabain, on the Na^+^,K^+^-pump in human mesenteric arteries. J. Hypertens. 16, 1953–1958. 10.1097/00004872-199816121-00015, PMID: 9886882

[ref14] Bagrov A. Y. Fedorova O. V. (2005). Cardenolide and bufadienolide ligands of the sodium pump. How they work together in NaCl sensitive hypertension. Front. Biosci. 10, 2250–2256. 10.2741/1694, PMID: 15970491

[ref15] Bagrov Y. Y. Manusova N. B. Egorova I. A. Fedorova O. V. Bagrov A. Y. (2005). Endogenous digitalis-like ligands and Na/K-ATPase inhibition in experimental diabetes mellitus. Front. Biosci. 10, 2257–2262. 10.2741/1695, PMID: 15970492

[ref16] Bagrov A. Y. Shapiro J. I. Fedorova O. V. (2009). Endogenous cardiotonic steroids: physiology, pharmacology, and novel therapeutic targets. Pharmacol. Rev. 61, 9–38. 10.1124/pr.108.000711, PMID: 19325075 PMC2763610

[ref17] Balaratnam S. Basu S. (2015). Divalent cation-aided identification of physico-chemical properties of metal ions that stabilize RNA G-quadruplexes. Biopolymers 103, 376–386. 10.1002/bip.22628, PMID: 25807937

[ref18] Barone F. C. White R. F. Spera P. A. Ellison J. Currie R. W. Wang X.. (1998). Ischemic preconditioning and brain tolerance: temporal histological and functional outcomes, protein synthesis requirement, and interleukin-1 receptor antagonist and early gene expression. Stroke 29, 1937–1950. 10.1161/01.STR.29.9.1937, PMID: 9731622

[ref19] Barros L. F. Hermosilla T. Castro J. (2001). Necrotic volume increase and the early physiology of necrosis. Comp. Biochem. Physiol. A Mol. Integr. Physiol. 130, 401–409. 10.1016/S1095-6433(01)00438-X, PMID: 11913453

[ref20] Blanco G. (2005). Na,K-ATPase subunit heterogeneity as a mechanism for tissue-specific ion regulation. Semin. Nephrol. 25, 292–303. 10.1016/j.semnephrol.2005.03.004, PMID: 16139684

[ref21] Blanco G. Mercer R. W. (1998). Isozymes of the Na-K-ATPase: heterogeneity in structure, diversity in function. Am. J. Physiol. 275, F633–F650. 10.1152/ajprenal.1998.275.5.F633, PMID: 9815123

[ref22] Blaustein M. P. (1996). Endogenous ouabain: role in the pathogenesis of hypertension. Kidney Int. 49, 1748–1753. 10.1038/ki.1996.260, PMID: 8743490

[ref23] Blaustein M. P. Hamlyn J. M. (2020). Ouabain, endogenous ouabain and ouabain-like factors: the Na^+^ pump/ouabain receptor, its linkage to NCX, and its myriad functions. Cell Calcium 86:102159. 10.1016/j.ceca.2020.102159, PMID: 31986323

[ref24] Bolívar J. J. Lázaro A. Fernández S. Stefani E. Peña-Cruz V. Lechene C.. (1987). Rescue of a wild-type MDCK cell by a ouabain-resistant mutant. Am. J. Physiol. 253, C151–C161. 10.1152/ajpcell.1987.253.1.C151, PMID: 3300360

[ref25] Bortner C. D. Cidlowski J. A. (1998). A necessary role for cell shrinkage in apoptosis. Biochem. Pharmacol. 56, 1549–1559. 10.1016/S0006-2952(98)00225-1, PMID: 9973175

[ref26] Bortner C. D. Cidlowski J. A. (2007). Cell shrinkage and monovalent cation fluxes: role in apoptosis. Arch. Biochem. Biophys. 462, 176–188. 10.1016/j.abb.2007.01.020, PMID: 17321483 PMC1941616

[ref27] Boutros T. Chevet E. Metrakos P. (2008). Mitogen-activated protein (MAP) kinase/MAP kinase phosphatase regulation: roles in cell growth, death, and cancer. Pharmacol. Rev. 60, 261–310. 10.1124/pr.107.00106, PMID: 18922965

[ref28] Burgos J. I. Morell M. Mariángelo J. I. E. Vila Petroff M. (2019). Hyperosmotic stress promotes endoplasmic reticulum stress-dependent apoptosis in adult rat cardiac myocytes. Apoptosis 24, 785–797. 10.1007/s10495-019-01558-4, PMID: 31309362

[ref29] Carini R. Autelli R. Bellomo G. Albano E. (1999). Alterations of cell volume regulation in the development of hepatocyte necrosis. Exp. Cell Res. 248, 280–293. 10.1006/excr.1999.4408, PMID: 10094834

[ref30] Carini R. Autelli R. Bellomo G. Dianzani M. U. Albano E. (1995). Sodium-mediated cell swelling is associated with irreversible damage in isolated hepatocytes exposed to hypoxia or mitochondrial toxins. Biochem. Biophys. Res. Commun. 206, 180–185. 10.1006/bbrc.1995.1025, PMID: 7818518

[ref31] Cheung J. Y. Zhang X. -Q. Song J. Gao E. Rabinowitz J. E. Chan T. O.. (2010). Review article: phospholemman: a novel cardiac stress protein. Clin. Transl. Sci. 3, 189–196. 10.1111/j.1752-8062.2010.00213.x, PMID: 20718822 PMC3013348

[ref32] Chou W. -H. Liu K. -L. Shih Y. -L. Chuang Y. -Y. Chou J. Lu H. -F.. (2018). Ouabain induces apoptotic cell death through caspase‐ and mitochondria-dependent pathways in human osteosarcoma U-2 OS cells. Anticancer Res. 38, 169–178. 10.21873/anticanres.12205, PMID: 29277770

[ref33] Chueh S. C. Guh J. H. Chen J. Lai M. K. Teng C. M. (2001). Dual effects of ouabain on the regulation of proliferation and apoptosis in human prostatic smooth muscle cells. J. Urol. 166, 347–353. 10.1016/S0022-5347(05)66157-5, PMID: 11435898

[ref34] Clausen M. V. Hilbers F. Poulsen H. (2017). The structure and function of the Na,K-ATPase isoforms in health and disease. Front. Physiol. 8:371. 10.3389/fphys.2017.00371, PMID: 28634454 PMC5459889

[ref35] Clouzeau C. Godefroy D. Riancho L. Rostène W. Baudouin C. Brignole-Baudouin F. (2012). Hyperosmolarity potentiates toxic effects of benzalkonium chloride on conjunctival epithelial cells in vitro. Mol. Vis. 18, 851–863. PMID: 22529703 PMC3332130

[ref36] Contreras R. G. Shoshani L. Flores-Maldonado C. Lázaro A. Cereijido M. (1999). Relationship between Na^+^,K^+^-ATPase and cell attachment. J. Cell Sci. 112, 4223–4232. PMID: 10564641 10.1242/jcs.112.23.4223

[ref37] Coulon V. Blanchard J. M. (2001). Flux calciques et expression génique. Med. Sci. 17, 969–978. 10.4267/10608/1810

[ref38] Cutaia M. Tollefson K. Kroczynski J. Parks N. Rounds S. (2000). Role of the Na/H antiport in pH-dependent cell death in pulmonary artery endothelial cells. Am. J. Physiol. Lung Cell. Mol. Physiol. 278, L536–L544. 10.1152/ajplung.2000.278.3.L536, PMID: 10710526

[ref39] D’Arcangelo D. Facchiano F. Barlucchi L. M. Melillo G. Illi B. Testolin L.. (2000). Acidosis inhibits endothelial cell apoptosis and function and induces basic fibroblast growth factor and vascular endothelial growth factor expression. Circ. Res. 86, 312–318. 10.1161/01.RES.86.3.312, PMID: 10679483

[ref40] D’Arcangelo D. Gaetano C. Capogrossi M. C. (2002). Acidification prevents endothelial cell apoptosis by Axl activation. Circ. Res. 91, e4–e12. 10.1161/01.res.0000036753.50601.e9, PMID: 12364394

[ref41] D’Arcy M. S. (2019). Cell death: a review of the major forms of apoptosis, necrosis and autophagy. Cell Biol. Int. 43, 582–592. 10.1002/cbin.11137, PMID: 30958602

[ref42] de Wardener H. E. (1996). Franz Volhard Lecture 1996. Sodium transport inhibitors and hypertension. J. Hypertens. Suppl. 14, S9–S18. PMID: 9120690

[ref43] Delebinski C. I. Georgi S. Kleinsimon S. Twardziok M. Kopp B. Melzig M. F.. (2015). Analysis of proliferation and apoptotic induction by 20 steroid glycosides in 143B osteosarcoma cells in vitro. Cell Prolif. 48, 600–610. 10.1111/cpr.12208, PMID: 26300346 PMC6496835

[ref44] Delpire E. Gagnon K. B. (2018). Water homeostasis and cell volume maintenance and regulation. Curr. Top. Membr. 81, 3–52. 10.1016/bs.ctm.2018.08.001, PMID: 30243436 PMC6457474

[ref45] Dmitrieva R. I. Doris P. A. (2002). Cardiotonic steroids: potential endogenous sodium pump ligands with diverse function. Exp. Biol. Med. 227, 561–569. 10.1177/153537020222700803, PMID: 12192097

[ref46] Dmitrieva N. I. Michea L. F. Rocha G. M. Burg M. B. (2001). Cell cycle delay and apoptosis in response to osmotic stress. Comp. Biochem. Physiol. A Mol. Integr. Physiol. 130, 411–420. 10.1016/S1095-6433(01)00439-1, PMID: 11913454

[ref47] Dorovkov M. V. Pavur K. S. Petrov A. N. Ryazanov A. G. (2002). Regulation of elongation factor-2 kinase by pH. Biochemistry 41, 13444–13450. 10.1021/bi026494p, PMID: 12416990

[ref48] Dostanic I. Lorenz J. N. Schultz J. E. J. Grupp I. L. Neumann J. C. Wani M. A.. (2003). The alpha2 isoform of Na,K-ATPase mediates ouabain-induced cardiac inotropy in mice. J. Biol. Chem. 278, 53026–53034. 10.1074/jbc.M308547200, PMID: 14559919

[ref49] Dostanic-Larson I. Van Huysse J. W. Lorenz J. N. Lingrel J. B. (2005). The highly conserved cardiac glycoside binding site of Na,K-ATPase plays a role in blood pressure regulation. Proc. Natl. Acad. Sci. U. S. A. 102, 15845–15850. 10.1073/pnas.0507358102, PMID: 16243970 PMC1276084

[ref50] Galluzzi L. Vitale I. Aaronson S. A. Abrams J. M. Adam D. Agostinis P.. (2018). Molecular mechanisms of cell death: recommendations of the Nomenclature Committee on Cell Death 2018. Cell Death Differ. 25, 486–541. 10.1038/s41418-017-0012-4, PMID: 29362479 PMC5864239

[ref51] Galvez A. S. Ulloa J. A. Chiong M. Criollo A. Eisner V. Barros L. F.. (2003). Aldose reductase induced by hyperosmotic stress mediates cardiomyocyte apoptosis: differential effects of sorbitol and mannitol. J. Biol. Chem. 278, 38484–38494. 10.1074/jbc.M211824200, PMID: 12881532

[ref52] Garty H. Karlish S. J. D. (2005). FXYD proteins: tissue-specific regulators of the Na,K-ATPase. Semin. Nephrol. 25, 304–311. 10.1016/j.semnephrol.2005.03.005, PMID: 16139685

[ref53] Geering K. (2001). The functional role of beta subunits in oligomeric P-type ATPases. J. Bioenerg. Biomembr. 33, 425–438. 10.1023/A:1010623724749, PMID: 11762918

[ref54] Geering K. (2005). Function of FXYD proteins, regulators of Na,K-ATPase. J. Bioenerg. Biomembr. 37, 387–392. 10.1007/s10863-005-9476-x, PMID: 16691470

[ref55] Geering K. (2008). Functional roles of Na,K-ATPase subunits. Curr. Opin. Nephrol. Hypertens. 17, 526–532. 10.1097/MNH.0b013e3283036cbf, PMID: 18695395

[ref56] Goto A. Ishiguro T. Yamada K. Ishii M. Yoshioka M. Eguchi C.. (1990). Isolation of a urinary digitalis-like factor indistinguishable from digoxin. Biochem. Biophys. Res. Commun. 173, 1093–1101. 10.1016/S0006-291X(05)80898-8, PMID: 2176483

[ref57] Grand C. L. Han H. Muñoz R. M. Weitman S. Von Hoff D. D. Hurley L. H.. (2002). The cationic porphyrin TMPyP4 down-regulates c-MYC and human telomerase reverse transcriptase expression and inhibits tumor growth in vivo. Mol. Cancer Ther. 1, 565–573. PMID: 12479216

[ref58] Groulx N. Boudreault F. Orlov S. N. Grygorczyk R. (2006). Membrane reserves and hypotonic cell swelling. J. Membr. Biol. 214, 43–56. 10.1007/s00232-006-0080-8, PMID: 17598067

[ref59] Gundersen K. (2011). Excitation-transcription coupling in skeletal muscle: the molecular pathways of exercise. Biol. Rev. Camb. Philos. Soc. 86, 564–600. 10.1111/j.1469-185X.2010.00161.x, PMID: 21040371 PMC3170710

[ref60] Haas M. Wang H. Tian J. Xie Z. (2002). Src-mediated inter-receptor cross-talk between the Na^+^/K^+^-ATPase and the epidermal growth factor receptor relays the signal from ouabain to mitogen-activated protein kinases. J. Biol. Chem. 277, 18694–18702. 10.1074/jbc.M111357200, PMID: 11907028

[ref61] Haloui M. Taurin S. Akimova O. A. Guo D. -F. Tremblay J. Dulin N. O.. (2007). [Na]_i_-induced c-Fos expression is not mediated by activation of the 5'-promoter containing known transcriptional elements. FEBS J. 274, 3557–3567. 10.1111/j.1742-4658.2007.05885.x, PMID: 17565602

[ref62] Hamlyn J. M. Manunta P. (2015). Endogenous cardiotonic steroids in kidney failure: a review and an hypothesis. Adv. Chronic Kidney Dis. 22, 232–244. 10.1053/j.ackd.2014.12.005, PMID: 25908473 PMC4410676

[ref63] Hardingham G. E. Chawla S. Johnson C. M. Bading H. (1997). Distinct functions of nuclear and cytoplasmic calcium in the control of gene expression. Nature 385, 260–265. 10.1038/385260a0, PMID: 9000075

[ref64] Hegyvary C. Jorgensen P. L. (1981). Conformational changes of renal sodium plus potassium ion-transport adenosine triphosphatase labeled with fluorescein. J. Biol. Chem. 256, 6296–6303. PMID: 6263913

[ref65] Hennion J. P. el-Masri M. A. Huff M. O. el-Mailakh R. S. (2002). Evaluation of neuroprotection by lithium and valproic acid against ouabain-induced cell damage. Bipolar Disord. 4, 201–206. 10.1034/j.1399-5618.2002.01162.x, PMID: 12180275

[ref66] Hoffmann E. K. Lambert I. H. Pedersen S. F. (2009). Physiology of cell volume regulation in vertebrates. Physiol. Rev. 89, 193–277. 10.1152/physrev.00037.2007, PMID: 19126758

[ref67] Hou X. Theriault S. F. Dostanic-Larson I. Moseley A. E. Lingrel J. B. Wu H.. (2009). Enhanced pressor response to increased CSF sodium concentration and to central ANG I in heterozygous alpha2 Na^+^-K^+^-ATPase knockout mice. Am. J. Physiol. Regul. Integr. Comp. Physiol. 296, R1427–R1438. 10.1152/ajpregu.00809.2007, PMID: 19244589 PMC2689841

[ref68] Isaev N. K. Stelmashook E. V. Halle A. Harms C. Lautenschlager M. Weih M.. (2000). Inhibition of Na^+^,K^+^-ATPase activity in cultured rat cerebellar granule cells prevents the onset of apoptosis induced by low potassium. Neurosci. Lett. 283, 41–44. 10.1016/S0304-3940(00)00903-4, PMID: 10729629

[ref69] Islam C. F. Mathie R. T. Dinneen M. D. Kiely E. A. Peters A. M. Grace P. A. (1997). Ischaemia-reperfusion injury in the rat kidney: the effect of preconditioning. Br. J. Urol. 79, 842–847. 10.1046/j.1464-410x.1997.00209.x, PMID: 9202547

[ref70] Kawamura A. Guo J. Itagaki Y. Bell C. Wang Y. Haupert G. T. (1999). On the structure of endogenous ouabain. Proc. Natl. Acad. Sci. U. S. A. 96, 6654–6659. 10.1073/pnas.96.12.6654 10359767 PMC21970

[ref71] Khalaf F. K. Dube P. Kleinhenz A. L. Malhotra D. Gohara A. Drummond C. A.. (2019). Proinflammatory effects of cardiotonic steroids mediated by NKA α-1 (Na^+^/K^+^-ATPase α-1)/Src complex in renal epithelial cells and immune cells. Hypertension 74, 73–82. 10.1161/HYPERTENSIONAHA.118.12605, PMID: 31132948 PMC6561827

[ref72] Khalaf F. K. Dube P. Mohamed A. Tian J. Malhotra D. Haller S. T.. (2018). Cardiotonic steroids and the sodium trade balance: new insights into trade-off mechanisms mediated by the Na^+^/K^+^-ATPase. Int. J. Mol. Sci. 19:2576. 10.3390/ijms19092576, PMID: 30200235 PMC6165267

[ref73] Kharel P. Balaratnam S. Beals N. Basu S. (2020). The role of RNA G-quadruplexes in human diseases and therapeutic strategies. Wiley Interdiscip. Rev. RNA 11:e1568. 10.1002/wrna.1568, PMID: 31514263

[ref74] Khundmiri S. J. (2014). Advances in understanding the role of cardiac glycosides in control of sodium transport in renal tubules. J. Endocrinol. 222, R11–R24. 10.1530/JOE-13-0613, PMID: 24781255

[ref75] Kim E. K. Choi E. -J. (2010). Pathological roles of MAPK signaling pathways in human diseases. Biochim. Biophys. Acta 1802, 396–405. 10.1016/j.bbadis.2009.12.009, PMID: 20079433

[ref184] Klimanova E. A. Petrushanko I. Y. Mitkevich V. A. Anashkina A. A. Orlov S. N. Makarov A. A.. (2015). Binding of ouabain and marinobufagenin leads to different structural changes in Na,K-ATPase and depends on the enzyme conformation. FEBS Lett. 589, 2668–2674. 10.1016/j.febslet.2015.08.011, PMID: 26297827

[ref76] Klimanova E. A. Sidorenko S. V. Smolyaninova L. V. Kapilevich L. V. Gusakova S. V. Lopina O. D.. (2019a). Ubiquitous and cell type-specific transcriptomic changes triggered by dissipation of monovalent cation gradients in rodent cells: physiological and pathophysiological implications. Curr. Top. Membr. 83, 107–149. 10.1016/bs.ctm.2019.01.006, PMID: 31196602

[ref77] Klimanova E. A. Sidorenko S. V. Tverskoi A. M. Shiyan A. A. Smolyaninova L. V. Kapilevich L. V.. (2019b). Search for intracellular sensors involved in the functioning of monovalent cations as secondary messengers. Biochem. Mosc. 84, 1280–1295. 10.1134/S0006297919110063, PMID: 31760918

[ref78] Klimanova E. A. Tverskoi A. M. Koltsova S. V. Sidorenko S. V. Lopina O. D. Tremblay J.. (2017). Time‐ and dose dependent actions of cardiotonic steroids on transcriptome and intracellular content of Na^+^ and K^+^: a comparative analysis. Sci. Rep. 7:45403. 10.1038/srep45403, PMID: 28345607 PMC5366943

[ref79] Koltsova S. V. Tremblay J. Hamet P. Orlov S. N. (2015). Transcriptomic changes in Ca^2+^-depleted cells: role of elevated intracellular [Na^+^]/[K^+^] ratio. Cell Calcium 58, 317–324. 10.1016/j.ceca.2015.06.009, PMID: 26183762

[ref80] Koltsova S. V. Trushina Y. Haloui M. Akimova O. A. Tremblay J. Hamet P.. (2012). Ubiquitous [Na^+^]_i_/[K^+^]_i_-sensitive transcriptome in mammalian cells: evidence for Ca(2+)_i_-independent excitation-transcription coupling. PLoS One 7:e38032. 10.1371/journal.pone.0038032, PMID: 22666440 PMC3362528

[ref81] Komiyama Y. Dong X. H. Nishimura N. Masaki H. Yoshika M. Masuda M.. (2005). A novel endogenous digitalis, telocinobufagin, exhibits elevated plasma levels in patients with terminal renal failure. Clin. Biochem. 38, 36–45. 10.1016/j.clinbiochem.2004.08.005, PMID: 15607315

[ref82] Koob R. Kraemer D. Trippe G. Aebi U. Drenckhahn D. (1990). Association of kidney and parotid Na^+^,K(+)-ATPase microsomes with actin and analogs of spectrin and ankyrin. Eur. J. Cell Biol. 53, 93–100. PMID: 1963843

[ref83] Kraemer D. Koob R. Friedrichs B. Drenckhahn D. (1990). Two novel peripheral membrane proteins, pasin 1 and pasin 2, associated with Na^+^,K(+)-ATPase in various cells and tissues. J. Cell Biol. 111, 2375–2383. 10.1083/jcb.111.6.2375, PMID: 2177475 PMC2116380

[ref84] Kraemer D. M. Strizek B. Meyer H. E. Marcus K. Drenckhahn D. (2003). Kidney Na^+^,K(+)-ATPase is associated with moesin. Eur. J. Cell Biol. 82, 87–92. 10.1078/0171-9335-00296, PMID: 12647934

[ref85] Krenn L. Kopp B. (1998). Bufadienolides from animal and plant sources. Phytochemistry 48, 1–29. 10.1016/S0031-9422(97)00426-3, PMID: 9621450

[ref86] Kulikov A. Eva A. Kirch U. Boldyrev A. Scheiner-Bobis G. (2007). Ouabain activates signaling pathways associated with cell death in human neuroblastoma. Biochim. Biophys. Acta 1768, 1691–1702. 10.1016/j.bbamem.2007.04.012, PMID: 17524349

[ref87] Kurosawa M. Tani Y. Nishimura S. Numazawa S. Yoshida T. (2001). Distinct PKC isozymes regulate bufalin-induced differentiation and apoptosis in human monocytic cells. Am. J. Physiol. Cell Physiol. 280, C459–C464. 10.1152/ajpcell.2001.280.3.C459, PMID: 11171564

[ref88] Lang F. Hoffmann E. K. (2012). Role of ion transport in control of apoptotic cell death. Compr. Physiol. 2, 2037–2061. 10.1002/cphy.c110046, PMID: 23723032

[ref89] Laursen M. Gregersen J. L. Yatime L. Nissen P. Fedosova N. U. (2015). Structures and characterization of digoxin‐ and bufalin-bound Na^+^,K^+^-ATPase compared with the ouabain-bound complex. Proc. Natl. Acad. Sci. U. S. A. 112, 1755–1760. 10.1073/pnas.1422997112, PMID: 25624492 PMC4330780

[ref90] Laursen M. Yatime L. Nissen P. Fedosova N. U. (2013). Crystal structure of the high-affinity Na^+^K^+^-ATPase-ouabain complex with Mg^2+^ bound in the cation binding site. Proc. Natl. Acad. Sci. U. S. A. 110, 10958–10963. 10.1073/pnas.1222308110, PMID: 23776223 PMC3704003

[ref91] Ledbetter M. L. Young G. J. Wright E. R. (1986). Cooperation between epithelial cells demonstrated by potassium transfer. Am. J. Physiol. 250, C306–C313. 10.1152/ajpcell.1986.250.2.C306, PMID: 3953783

[ref92] Lee J. M. Grabb M. C. Zipfel G. J. Choi D. W. (2000). Brain tissue responses to ischemia. J. Clin. Invest. 106, 723–731. 10.1172/JCI11003, PMID: 10995780 PMC381398

[ref93] Lee K. Jung J. Kim M. Guidotti G. (2001). Interaction of the alpha subunit of Na,K-ATPase with cofilin. Biochem. J. 353, 377–385. 10.1042/bj3530377, PMID: 11139403 PMC1221581

[ref94] Li Q. Chen M. Liu H. Yang L. Yang T. He G. (2014). The dual role of ERK signaling in the apoptosis of neurons. Front. Biosci. 19, 1411–1417. 10.2741/4291, PMID: 24896360

[ref95] Li Y. Tian X. Liu X. Gong P. (2018). Bufalin inhibits human breast cancer tumorigenesis by inducing cell death through the ROS-mediated RIP1/RIP3/PARP-1 pathways. Carcinogenesis 39, 700–707. 10.1093/carcin/bgy039, PMID: 29546393

[ref96] Li Y. W. Whittaker P. Kloner R. A. (1992). The transient nature of the effect of ischemic preconditioning on myocardial infarct size and ventricular arrhythmia. Am. Heart J. 123, 346–353. 10.1016/0002-8703(92)90645-C, PMID: 1736569

[ref97] Lichtstein D. Gati I. Samuelov S. Berson D. Rozenman Y. Landau L.. (1993). Identification of digitalis-like compounds in human cataractous lenses. Eur. J. Biochem. 216, 261–268. 10.1111/j.1432-1033.1993.tb18141.x, PMID: 8396030

[ref98] Lingrel J. B. (2010). The physiological significance of the cardiotonic steroid/ouabain-binding site of the Na,K-ATPase. Annu. Rev. Physiol. 72, 395–412. 10.1146/annurev-physiol-021909-135725, PMID: 20148682 PMC3079441

[ref99] Lingrel J. B. Argüello J. M. Van Huysse J. Kuntzweiler T. A. (1997). Cation and cardiac glycoside binding sites of the Na,K-ATPase. Ann. N. Y. Acad. Sci. 834, 194–206. 10.1111/j.1749-6632.1997.tb52251.x, PMID: 9405808

[ref100] Lingrel J. B. Williams M. T. Vorhees C. V. Moseley A. E. (2007). Na,K-ATPase and the role of alpha isoforms in behavior. J. Bioenerg. Biomembr. 39, 385–389. 10.1007/s10863-007-9107-9, PMID: 18044013

[ref101] Liu C. Bai Y. Chen Y. Wang Y. Sottejeau Y. Liu L.. (2012). Reduction of Na/K-ATPase potentiates marinobufagenin-induced cardiac dysfunction and myocyte apoptosis. J. Biol. Chem. 287, 16390–16398. 10.1074/jbc.M111.304451, PMID: 22451662 PMC3351339

[ref102] Liu J. Kesiry R. Periyasamy S. M. Malhotra D. Xie Z. Shapiro J. I. (2004). Ouabain induces endocytosis of plasmalemmal Na/K-ATPase in LLC-PK1 cells by a clathrin-dependent mechanism. Kidney Int. 66, 227–241. 10.1111/j.1523-1755.2004.00723.x, PMID: 15200429

[ref103] Liu J. Xie Z. -J. (2010). The sodium pump and cardiotonic steroids-induced signal transduction protein kinases and calcium-signaling microdomain in regulation of transporter trafficking. Biochim. Biophys. Acta 1802, 1237–1245. 10.1016/j.bbadis.2010.01.013, PMID: 20144708 PMC5375027

[ref104] Lopatin D. A. Ailamazian E. K. Dmitrieva R. I. Shpen V. M. Fedorova O. V. Doris P. A.. (1999). Circulating bufodienolide and cardenolide sodium pump inhibitors in preeclampsia. J. Hypertens. 17, 1179–1187. 10.1097/00004872-199917080-00018, PMID: 10466474

[ref105] Loreaux E. L. Kaul B. Lorenz J. N. Lingrel J. B. (2008). Ouabain-sensitive alpha1 Na,K-ATPase enhances natriuretic response to saline load. J. Am. Soc. Nephrol. 19, 1947–1954. 10.1681/ASN.2008020174, PMID: 18667729 PMC2551575

[ref106] Ma H. Groth R. D. Wheeler D. G. Barrett C. F. Tsien R. W. (2011). Excitation-transcription coupling in sympathetic neurons and the molecular mechanism of its initiation. Neurosci. Res. 70, 2–8. 10.1016/j.neures.2011.02.004, PMID: 21352861 PMC3930329

[ref107] Maingret F. Patel A. J. Lesage F. Lazdunski M. Honoré E. (1999). Mechano‐ or acid stimulation, two interactive modes of activation of the TREK-1 potassium channel. J. Biol. Chem. 274, 26691–26696. 10.1074/jbc.274.38.26691, PMID: 10480871

[ref108] Majno G. Joris I. (1995). Apoptosis, oncosis, and necrosis. An overview of cell death. Am. J. Pathol. 146, 3–15. PMID: 7856735 PMC1870771

[ref109] Matsuyama S. Reed J. C. (2000). Mitochondria-dependent apoptosis and cellular pH regulation. Cell Death Differ. 7, 1155–1165. 10.1038/sj.cdd.4400779, PMID: 11175252

[ref110] Matthews C. C. Feldman E. L. (1996). Insulin-like growth factor I rescues SH-SY5Y human neuroblastoma cells from hyperosmotic induced programmed cell death. J. Cell. Physiol. 166, 323–331. 10.1002/(SICI)1097-4652(199602)166:2<323::AID-JCP10>3.0.CO;2-C, PMID: 8591992

[ref111] McConkey D. J. Lin Y. Nutt L. K. Ozel H. Z. Newman R. A. (2000). Cardiac glycosides stimulate Ca^2+^ increases and apoptosis in androgen-independent, metastatic human prostate adenocarcinoma cells. Cancer Res. 60, 3807–3812. PMID: 10919654

[ref112] McDonald T. F. Pelzer S. Trautwein W. Pelzer D. J. (1994). Regulation and modulation of calcium channels in cardiac, skeletal, and smooth muscle cells. Physiol. Rev. 74, 365–507. 10.1152/physrev.1994.74.2.365, PMID: 8171118

[ref113] Meng L. Wen Y. Zhou M. Li J. Wang T. Xu P.. (2016). Ouabain induces apoptosis and autophagy in Burkitt’s lymphoma Raji cells. Biomed. Pharmacother. 84, 1841–1848. 10.1016/j.biopha.2016.10.114, PMID: 27894666

[ref114] Miyakawa-Naito A. Uhlén P. Lal M. Aizman O. Mikoshiba K. Brismar H.. (2003). Cell signaling microdomain with Na,K-ATPase and inositol 1,4,5-trisphosphate receptor generates calcium oscillations. J. Biol. Chem. 278, 50355–50361. 10.1074/jbc.M305378200, PMID: 12947118

[ref115] Mongin A. A. Orlov S. N. (2001). Mechanisms of cell volume regulation and possible nature of the cell volume sensor. Pathophysiology 8, 77–88. 10.1016/S0928-4680(01)00074-8, PMID: 11720802

[ref116] Morrow J. S. Cianci C. D. Ardito T. Mann A. S. Kashgarian M. (1989). Ankyrin links fodrin to the alpha subunit of Na,K-ATPase in Madin-Darby canine kidney cells and in intact renal tubule cells. J. Cell Biol. 108, 455–465. 10.1083/jcb.108.2.455, PMID: 2537316 PMC2115445

[ref117] Munshi A. Ramesh R. (2013). Mitogen-activated protein kinases and their role in radiation response. Genes Cancer 4, 401–408. 10.1177/1947601913485414, PMID: 24349638 PMC3863336

[ref118] Ogawa H. Shinoda T. Cornelius F. Toyoshima C. (2009). Crystal structure of the sodium-potassium pump (Na^+^,K^+^-ATPase) with bound potassium and ouabain. Proc. Natl. Acad. Sci. U. S. A. 106, 13742–13747. 10.1073/pnas.0907054106, PMID: 19666591 PMC2728964

[ref119] Okada Y. Maeno E. Shimizu T. Dezaki K. Wang J. Morishima S. (2001). Receptor-mediated control of regulatory volume decrease (RVD) and apoptotic volume decrease (AVD). J. Physiol. 532, 3–16. 10.1111/j.1469-7793.2001.0003g.x, PMID: 11283221 PMC2278524

[ref120] Orlov S. N. Dam T. V. Tremblay J. Hamet P. (1996). Apoptosis in vascular smooth muscle cells: role of cell shrinkage. Biochem. Biophys. Res. Commun. 221, 708–715. 10.1006/bbrc.1996.0661, PMID: 8630026

[ref121] Orlov S. N. Hamet P. (2004). Apoptosis vs. oncosis: role of cell volume and intracellular monovalent cations. Adv. Exp. Med. Biol. 559, 219–233. 10.1007/0-387-23752-6_21, PMID: 18727243

[ref122] Orlov S. N. Hamet P. (2006). The death of cardiotonic steroid-treated cells: evidence of Na^+^_i_,K^+^_i_-independent H^+^_i_-sensitive signalling. Acta Physiol. 187, 231–240. 10.1111/j.1748-1716.2006.01546.x, PMID: 16734760

[ref123] Orlov S. N. Hamet P. (2015). Salt and gene expression: evidence for [Na^+^]_i_/[K^+^]_i_-mediated signaling pathways. Pflugers Arch. 467, 489–498. 10.1007/s00424-014-1650-8, PMID: 25479826

[ref124] Orlov S. N. Klimanova E. A. Tverskoi A. M. Vladychenskaya E. A. Smolyaninova L. V. Lopina O. D. (2017). Na^+^_i_,K^+^_i_-dependent and -independent signaling triggered by cardiotonic steroids: facts and artifacts. Molecules 22:635. 10.3390/molecules22040635, PMID: 28420099 PMC6153942

[ref125] Orlov S. N. Pchejetski D. Taurin S. Thorin-Trescases N. Maximov G. V. Pshezhetsky A. V.. (2004a). Apoptosis in serum-deprived vascular smooth muscle cells: evidence for cell volume-independent mechanism. Apoptosis 9, 55–66. 10.1023/B:APPT.0000012122.47197.03, PMID: 14739599

[ref126] Orlov S. N. Taurin S. Tremblay J. Hamet P. (2001). Inhibition of Na^+^,K^+^ pump affects nucleic acid synthesis and smooth muscle cell proliferation via elevation of the [Na^+^]_i_/[K^+^]_i_ ratio: possible implication in vascular remodelling. J. Hypertens. 19, 1559–1565. 10.1097/00004872-200109000-00007, PMID: 11564975

[ref127] Orlov S. N. Thorin-Trescases N. Kotelevtsev S. V. Tremblay J. Hamet P. (1999). Inversion of the intracellular Na^+^/K^+^ ratio blocks apoptosis in vascular smooth muscle at a site upstream of caspase-3. J. Biol. Chem. 274, 16545–16552. 10.1074/jbc.274.23.16545, PMID: 10347219

[ref128] Orlov S. N. Thorin-Trescases N. Pchejetski D. Taurin S. Farhat N. Tremblay J.. (2004b). Na^+^/K^+^ pump and endothelial cell survival: [Na^+^]_i_/[K_+_]_i_-independent necrosis triggered by ouabain, and protection against apoptosis mediated by elevation of [Na^+^]_i_. Pflugers Arch. 448, 335–345. 10.1007/s00424-004-1262-9, PMID: 15069561

[ref129] Orlov S. N. Tverskoi A. M. Sidorenko S. V. Smolyaninova L. V. Lopina O. D. Dulin N. O. (2020). Na,K-ATPase as a target for endogenous cardiotonic steroids: what’s the evidence? Genes Dis. 10.1016/j.gendis.2020.01.008 (in press). PMC8093582 33997173

[ref130] Özdemir A. Şimay Y. D. İbişoğlu B. Yaren B. Bülbül D. Ark M. (2016). Cardiac glycoside-induced cell death and Rho/Rho kinase pathway: implication of different regulation in cancer cell lines. Steroids 109, 29–43. 10.1016/j.steroids.2016.03.015, PMID: 27017918

[ref131] Paczula A. Więcek A. Piecha G. (2016). The role of endogenous cardiotonic steroids in pathogenesis of cardiovascular and renal complications of arterial hypertension. Postepy Hig. Med. Dosw. 70, 243–250. 10.5604/17322693.1197486, PMID: 27117099

[ref132] Pasantes-Morales H. (2016). Channels and volume changes in the life and death of the cell. Mol. Pharmacol. 90, 358–370. 10.1124/mol.116.104158, PMID: 27358231

[ref133] Pavlovic D. (2014). The role of cardiotonic steroids in the pathogenesis of cardiomyopathy in chronic kidney disease. Nephron Clin. Pract. 128, 11–21. 10.1159/000363301, PMID: 25341357

[ref134] Pchejetski D. Taurin S. Der Sarkissian S. Lopina O. D. Pshezhetsky A. V. Tremblay J.. (2003). Inhibition of Na^+^,K^+^-ATPase by ouabain triggers epithelial cell death independently of inversion of the [Na^+^]i/[K^+^]_i_ ratio. Biochem. Biophys. Res. Commun. 301, 735–744. 10.1016/S0006-291X(02)03002-4, PMID: 12565842

[ref135] Pérez-Sala D. Collado-Escobar D. Mollinedo F. (1995). Intracellular alkalinization suppresses lovastatin-induced apoptosis in HL-60 cells through the inactivation of a pH-dependent endonuclease. J. Biol. Chem. 270, 6235–6242. 10.1074/jbc.270.11.6235, PMID: 7890761

[ref136] Perne A. Muellner M. K. Steinrueck M. Craig-Mueller N. Mayerhofer J. Schwarzinger I.. (2009). Cardiac glycosides induce cell death in human cells by inhibiting general protein synthesis. PLoS One 4:e8292. 10.1371/journal.pone.0008292, PMID: 20016840 PMC2788214

[ref137] Peters A. A. Jamaludin S. Y. N. Yapa K. T. D. S. Chalmers S. Wiegmans A. P. Lim H. F.. (2017). Oncosis and apoptosis induction by activation of an overexpressed ion channel in breast cancer cells. Oncogene 36, 6490–6500. 10.1038/onc.2017.234, PMID: 28759041

[ref138] Pezzani R. Rubin B. Redaelli M. Radu C. Barollo S. Cicala M. V.. (2014). The antiproliferative effects of ouabain and everolimus on adrenocortical tumor cells. Endocr. J. 61, 41–53. 10.1507/endocrj.EJ13-0225, PMID: 24153038

[ref139] Piper A. S. Large W. A. (2003). Multiple conductance states of single Ca^2+^-activated Cl-channels in rabbit pulmonary artery smooth muscle cells. J. Physiol. 547, 181–196. 10.1113/jphysiol.2002.033688, PMID: 12562904 PMC2342635

[ref140] Platonova A. Koltsova S. Maksimov G. V. Grygorczyk R. Orlov S. N. (2011). The death of ouabain-treated renal epithelial C11-MDCK cells is not mediated by swelling-induced plasma membrane rupture. J. Membr. Biol. 241, 145–154. 10.1007/s00232-011-9371-9, PMID: 21584679

[ref141] Png E. Samivelu G. K. Yeo S. H. Chew J. Chaurasia S. S. Tong L. (2011). Hyperosmolarity-mediated mitochondrial dysfunction requires Transglutaminase-2 in human corneal epithelial cells. J. Cell. Physiol. 226, 693–699. 10.1002/jcp.22389, PMID: 20717931

[ref142] Radzyukevich T. L. Lingrel J. B. Heiny J. A. (2009). The cardiac glycoside binding site on the Na,K-ATPase α2 isoform plays a role in the dynamic regulation of active transport in skeletal muscle. Proc. Natl. Acad. Sci. U. S. A. 106, 2565–2570. 10.1073/pnas.0804150106, PMID: 19196986 PMC2650304

[ref143] Rajan S. Wischmeyer E. Xin Liu G. Preisig-Müller R. Daut J. Karschin A.. (2000). TASK-3, a novel tandem pore domain acid-sensitive K+ channel. An extracellular histiding as pH sensor. J. Biol. Chem. 275, 16650–16657. 10.1074/jbc.M000030200, PMID: 10747866

[ref144] Rajasekaran S. A. Gopal J. Rajasekaran A. K. (2003). Expression of Na,K-ATPase beta-subunit in transformed MDCK cells increases the translation of the Na,K-ATPase alpha-subunit. Ann. N. Y. Acad. Sci. 986, 652–654. 10.1111/j.1749-6632.2003.tb07277.x, PMID: 12763913

[ref145] Ravichandran S. Ahn J. -H. Kim K. K. (2019). Unraveling the regulatory G-quadruplex puzzle: lessons from genome and transcriptome-wide studies. Front. Genet. 10:1002. 10.3389/fgene.2019.01002, PMID: 31681431 PMC6813735

[ref146] Reinehr R. Häussinger D. (2006). Hyperosmotic activation of the CD95 death receptor system. Acta Physiol. 187, 199–203. 10.1111/j.1748-1716.2006.01541.x, PMID: 16734756

[ref147] Riganti C. Campia I. Kopecka J. Gazzano E. Doublier S. Aldieri E.. (2011). Pleiotropic effects of cardioactive glycosides. Curr. Med. Chem. 18, 872–885. 10.2174/092986711794927685, PMID: 21182478

[ref148] Rosen H. Glukhman V. Feldmann T. Fridman E. Lichtstein D. (2004). Cardiac steroids induce changes in recycling of the plasma membrane in human NT2 cells. Mol. Biol. Cell 15, 1044–1054. 10.1091/mbc.e03-06-0391, PMID: 14718569 PMC363072

[ref149] Ryazanov A. G. (2002). Elongation factor-2 kinase and its newly discovered relatives. FEBS Lett. 514, 26–29. 10.1016/S0014-5793(02)02299-8, PMID: 11904175

[ref150] Ryazanov A. G. Shestakova E. A. Natapov P. G. (1988). Phosphorylation of elongation factor 2 by EF-2 kinase affects rate of translation. Nature 334, 170–173. 10.1038/334170a0, PMID: 3386756

[ref151] Schatzmann H. J. (1953). Cardiac glycosides as inhibitors of active potassium and sodium transport by erythrocyte membrane. Helv. Physiol. Pharmacol. Acta 11, 346–354. PMID: 13142506

[ref152] Scheiner-Bobis G. (2002). The sodium pump. Eur. J. Biochem. 269, 2424–2433. 10.1046/j.1432-1033.2002.02909.x, PMID: 12027879

[ref153] Schneider R. Antolovic R. Kost H. Sich B. Kirch U. Tepel M.. (1998a). Proscillaridin a immunoreactivity: its purification, transport in blood by a specific binding protein and its correlation with blood pressure. Clin. Exp. Hypertens. 20, 593–599. 10.3109/10641969809053237, PMID: 9682915

[ref154] Schneider R. Wray V. Nimtz M. Lehmann W. D. Kirch U. Antolovic R.. (1998b). Bovine adrenals contain, in addition to ouabain, a second inhibitor of the sodium pump. J. Biol. Chem. 273, 784–792. 10.1074/jbc.273.2.784, PMID: 9422732

[ref155] Schoner W. (2002). Endogenous cardiac glycosides, a new class of steroid hormones. Eur. J. Biochem. 269, 2440–2448. 10.1046/j.1432-1033.2002.02911.x, PMID: 12027881

[ref156] Shinoda T. Ogawa H. Cornelius F. Toyoshima C. (2009). Crystal structure of the sodium-potassium pump at 2.4 A resolution. Nature 459, 446–450. 10.1038/nature07939, PMID: 19458722

[ref157] Siesjö B. K. Katsura K. Kristián T. (1996). Acidosis-related damage. Adv. Neurol. 71, 209–233. PMID: 8790801

[ref158] Skou J. C. (1960). Further investigations on a Mg^++^ + Na^+^-activated adenosintriphosphatase, possibly related to the active, linked transport of Na^+^ and K^+^ across the nerve membrane. Biochim. Biophys. Acta 42, 6–23. 10.1016/0006-3002(60)90746-0

[ref159] Smolyaninova L. V. Koltsova S. V. Sidorenko S. V. Orlov S. N. (2017). Augmented gene expression triggered by Na^+^,K^+^-ATPase inhibition: role of Ca^2+^_i_-mediated and -independent excitation-transcription coupling. Cell Calcium 68, 5–13. 10.1016/j.ceca.2017.10.002, PMID: 29129208

[ref160] Smolyaninova L. V. Shiyan A. A. Kapilevich L. V. Lopachev A. V. Fedorova T. N. Klementieva T. S.. (2019). Transcriptomic changes triggered by ouabain in rat cerebellum granule cells: role of α3‐ and α1-Na^+^,K^+^-ATPase-mediated signaling. PLoS One 14:e0222767. 10.1371/journal.pone.0222767, PMID: 31557202 PMC6762055

[ref161] Taurin S. Dulin N. O. Pchejetski D. Grygorczyk R. Tremblay J. Hamet P.. (2002a). c-Fos expression in ouabain-treated vascular smooth muscle cells from rat aorta: evidence for an intracellular-sodium-mediated, calcium-independent mechanism. J. Physiol. 543, 835–847. 10.1113/jphysiol.2002.023259, PMID: 12231642 PMC2290551

[ref162] Taurin S. Seyrantepe V. Orlov S. N. Tremblay T. -L. Thibault P. Bennett M. R.. (2002b). Proteome analysis and functional expression identify mortalin as an antiapoptotic gene induced by elevation of [Na^+^]_i_/[K^+^]_i_ ratio in cultured vascular smooth muscle cells. Circ. Res. 91, 915–922. 10.1161/01.res.0000043020.45534.3e, PMID: 12433836

[ref163] Terada Y. Inoshita S. Hanada S. Shimamura H. Kuwahara M. Ogawa W.. (2001). Hyperosmolality activates Akt and regulates apoptosis in renal tubular cells. Kidney Int. 60, 553–567. 10.1046/j.1523-1755.2001.060002553.x, PMID: 11473638

[ref164] Thornton T. M. Rincon M. (2009). Non-classical p38 map kinase functions: cell cycle checkpoints and survival. Int. J. Biol. Sci. 5, 44–51. 10.7150/ijbs.5.44, PMID: 19159010 PMC2610339

[ref165] Trenti A. Grumati P. Cusinato F. Orso G. Bonaldo P. Trevisi L. (2014). Cardiac glycoside ouabain induces autophagic cell death in non-small cell lung cancer cells via a JNK-dependent decrease of Bcl-2. Biochem. Pharmacol. 89, 197–209. 10.1016/j.bcp.2014.02.021, PMID: 24630927

[ref166] Triana-Martínez F. Picallos-Rabina P. Da Silva-Álvarez S. Pietrocola F. Llanos S. Rodilla V.. (2019). Identification and characterization of cardiac glycosides as senolytic compounds. Nat. Commun. 10:4731. 10.1038/s41467-019-12888-x, PMID: 31636264 PMC6803708

[ref167] Tverskoi A. M. Lokteva V. A. Orlov S. N. Lopina O. D. (2020). Binding of ouabain, digoxin, or marinobufagenin induces different conformational changes in kidney α1-Na^+^,K^+^-ATPase isoforms, resistant and sensitive to cardiotonic steroids. Biochem. Moscow Suppl. Ser. A 14, 54–60. 10.1134/S1990747820010080

[ref168] Van Cruchten S. Van den Broeck W. (2002). Morphological and biochemical aspects of apoptosis, oncosis and necrosis. Anat. Histol. Embryol. 31, 214–223. 10.1046/j.1439-0264.2002.00398.x, PMID: 12196263

[ref169] Verma A. Halder K. Halder R. Yadav V. K. Rawal P. Thakur R. K.. (2008). Genome-wide computational and expression analyses reveal G-quadruplex DNA motifs as conserved cis-regulatory elements in human and related species. J. Med. Chem. 51, 5641–5649. 10.1021/jm800448a, PMID: 18767830

[ref170] Wang H. Haas M. Liang M. Cai T. Tian J. Li S.. (2004). Ouabain assembles signaling cascades through the caveolar Na^+^/K^+^-ATPase. J. Biol. Chem. 279, 17250–17259. 10.1074/jbc.M313239200, PMID: 14963033

[ref171] Ward S. C. Hamilton B. P. Hamlyn J. M. (2002). Novel receptors for ouabain: studies in adrenocortical cells and membranes. Hypertension 39, 536–542. 10.1161/hy0202.103048, PMID: 11882604

[ref172] Weerasinghe P. Buja L. M. (2012). Oncosis: an important non-apoptotic mode of cell death. Exp. Mol. Pathol. 93, 302–308. 10.1016/j.yexmp.2012.09.018, PMID: 23036471

[ref173] Williams R. S. Benjamin I. J. (2000). Protective responses in the ischemic myocardium. J. Clin. Invest. 106, 813–818. 10.1172/JCI11205, PMID: 11018066 PMC381426

[ref174] Withering W. (1785). An account of the foxglove, and some of its medical uses: With practical remarks on dropsy and other diseases. Birmingham, England: Classics of Medicine Library.

[ref175] Włodarczyk A. Grzybowski P. Patkowski A. Dobek A. (2005). Effect of ions on the polymorphism, effective charge, and stability of human telomeric DNA. Photon correlation spectroscopy and circular dichroism studies. J. Phys. Chem. B 109, 3594–3605. 10.1021/jp045274d, PMID: 16851398

[ref176] Xie Z. Askari A. (2002). Na(+)/K(+)-ATPase as a signal transducer. Eur. J. Biochem. 269, 2434–2439. 10.1046/j.1432-1033.2002.02910.x, PMID: 12027880

[ref177] Xie Z. Cai T. (2003). Na^+^-K^+^-ATPase-mediated signal transduction: from protein interaction to cellular function. Mol. Interv. 3, 157–168. 10.1124/mi.3.3.157, PMID: 14993422

[ref178] Xiong Z. -G. Zhu X. -M. Chu X. -P. Minami M. Hey J. Wei W. -L.. (2004). Neuroprotection in ischemia: blocking calcium-permeable acid-sensing ion channels. Cell 118, 687–698. 10.1016/j.cell.2004.08.026, PMID: 15369669

[ref179] Yamaguchi M. Tonomura Y. (1979). Simultaneous binding of three Na_+_ and two K^+^ ions to Na^+^,K^+^-dependent ATPase and changes in its affinities for the ions induced by the formation of a phosphorylated intermediate. J. Biochem. 86, 509–523. 10.1093/oxfordjournals.jbchem.a132551, PMID: 225309

[ref180] Yatime L. Laursen M. Morth J. P. Esmann M. Nissen P. Fedosova N. U. (2011). Structural insights into the high affinity binding of cardiotonic steroids to the Na^+^,K^+^-ATPase. J. Struct. Biol. 174, 296–306. 10.1016/j.jsb.2010.12.004, PMID: 21182963

[ref181] Yudowski G. A. Efendiev R. Pedemonte C. H. Katz A. I. Berggren P. O. Bertorello A. M. (2000). Phosphoinositide-3 kinase binds to a proline-rich motif in the Na^+^,K^+^-ATPase alpha subunit and regulates its trafficking. Proc. Natl. Acad. Sci. U. S. A. 97, 6556–6561. 10.1073/pnas.100128297, PMID: 10823893 PMC18657

[ref182] Yurinskaya V. Goryachaya T. Guzhova I. Moshkov A. Rozanov Y. Sakuta G.. (2005). Potassium and sodium balance in U937 cells during apoptosis with and without cell shrinkage. CPB 16, 155–162. 10.1159/000089841, PMID: 16301816

[ref183] Zhou X. Jiang G. Zhao A. Bondeva T. Hirszel P. Balla T. (2001). Inhibition of Na,K-ATPase activates PI3 kinase and inhibits apoptosis in LLC-PK1 cells. Biochem. Biophys. Res. Commun. 285, 46–51. 10.1006/bbrc.2001.5126, PMID: 11437370

